# Racism and health in New Zealand: Prevalence over time and associations between recent experience of racism and health and wellbeing measures using national survey data

**DOI:** 10.1371/journal.pone.0196476

**Published:** 2018-05-03

**Authors:** Ricci B. Harris, James Stanley, Donna M. Cormack

**Affiliations:** 1 Eru Pōmare Māori Health Research Centre, Department of Public Health, University of Otago, Wellington, New Zealand; 2 Dean’s Department, University of Otago, Wellington, New Zealand; Universidad del Desarrollo, CHILE

## Abstract

**Objectives:**

Racism is an important health determinant that contributes to ethnic health inequities. This study sought to describe New Zealand adults’ reported recent experiences of racism over a 10 year period. It also sought to examine the association between recent experience of racism and a range of negative health and wellbeing measures.

**Methods:**

The study utilised previously collected data from multiple cross-sectional national surveys (New Zealand Health Surveys 2002/03, 2006/07, 2011/12; and General Social Surveys 2008, 2010, 2012) to provide prevalence estimates of reported experience of racism (in the last 12 months) by major ethnic groupings in New Zealand. Meta-analytical techniques were used to provide improved estimates of the association between recent experience of racism and negative health from multivariable models, for the total cohorts and stratified by ethnicity.

**Results:**

Reported recent experience of racism was highest among Asian participants followed by Māori and Pacific peoples, with Europeans reporting the lowest experience of racism. Among Asian participants, reported experience of racism was higher for those born overseas compared to those born in New Zealand. Recent experience of racism appeared to be declining for most groups over the time period examined. Experience of racism in the last 12 months was consistently associated with negative measures of health and wellbeing (SF-12 physical and mental health component scores, self-rated health, overall life satisfaction). While exposure to racism was more common in the non-European ethnic groups, the impact of recent exposure to racism on health was similar across ethnic groups, with the exception of SF-12 physical health.

**Conclusions:**

The higher experience of racism among non-European groups remains an issue in New Zealand and its potential effects on health may contribute to ethnic health inequities. Ongoing focus and monitoring of racism as a determinant of health is required to inform and improve interventions.

## Introduction

Racism has been established as an important determinant of health and a root cause of racial/ethnic health inequities [1]. Racism can be understood as an organised system with historical contexts and contemporary manifestations [1, 2]. It involves categorising and ranking racial/ethnic groups into social hierarchies, whereby racial/ethnic groups are assigned differential value and have differential access to power, opportunities and resources [1]. There are many expressions of racism at structural and individual levels that can affect health through a number of pathways [1].

There has been considerable growth over the last two decades in research on the negative impacts of racism on health, particularly with regards to the impact of experience of interpersonal racism [3]. There is now a strong, consistent evidence base linking experience of racial discrimination to various markers of negative physical and mental health (both self-reported and objectively measured), health risk and health care utilisation. The magnitude of association appears to be highest with mental health outcomes [3]. Research has been predominantly carried out among adult population samples [1, 3–5], although there is increasing research interest in examining experiences of racism and links to health for children [6]. Research among adults also extends into evidence for associations between experience of racism and broader measures of wellbeing such as life satisfaction, quality of life and self-esteem [5, 7–11]. While most studies are cross-sectional, associations between experience of racism and negative health measures are also evident in longitudinal studies [3, 4].

In New Zealand (NZ), there are long-standing and systematic ethnic inequities in health outcomes, risk factors (including broader social determinants) and health care, particularly between Māori (the indigenous peoples) and European ethnic groups [12–14]. Population level studies have demonstrated higher prevalence of reported experience of racism by non-European ethnic groups, particularly Asian and Māori [15–20]. A growing body of local research shows a consistent link between experience of racism and a range of negative health measures that may significantly impact on ethnic health inequities [15–19, 21–26]. This includes studies investigating a range of mental and physical health measures, individual level risk factors and health service experience and use. Analysis of data from the adult New Zealand Health Surveys (NZHS 2002/03 and 2006/07) showed that reported experience of racial discrimination ‘ever’ was significantly associated with a range of negative health measures for all ethnic groups, including self-rated general health, self-rated physical functioning and mental health, psychological distress, diagnosed cardiovascular disesase, diagnosed mental health disorder, smoking and sleep disturbance [16, 17, 26]. Both the experience of interpersonal racism and socioeconomic position (as a marker of systemic racism) have been shown to contribute to health inequities between Māori and European ethnic groups [18]. Analysis from the 2006/07 NZHS also showed that experience of racial discrimination was associated with less positive patient experiences with their usual primary care provider and, for Māori women, lower breast and cervical cancer screening coverage [22].

Longitudinal evidence for these links is also available from New Zealand. The NZ Attitudes and Values study has shown that experience of racism was negatively linked to subsequent wellbeing among Māori [24]. The Growing Up in New Zealand study demonstrated that experience of racism (as reported during pregnancy) was linked to higher likelihood of postnatal depression among Māori, Pacific and Asian women [19]. Experiences of racism have also been more broadly documented in other New Zealand studies, both quantitative and qualitative [27–33], including studies finding negative attitudes and stereotypes towards Māori by health providers [34–37].

These studies provide an important local evidence base to build upon in order to better understand and address racism as a determinant of health in New Zealand. To date, local studies have tended to focus on a relatively small pool of exposed individuals (giving a small effective sample size) or limited study time periods. These studies can consequently be limited in their power to examine relationships between recent experience of racism (last 12 months) and health, particularly for specific ethnic groups. In addition, there is limited international evidence of the relationship between racism and health for individual ethnic groups, although existing research suggests that the strength of association may differ by race/ethnicity and by time from exposure to outcome [3].

The routine monitoring of experience of racism as a determinant of health and wellbeing has now been implemented in multiple time periods for two of our national surveys, the New Zealand Health Survey (2002/03, 2006/07, 2011/12) and the General Social Survey (GSS 2008, GSS 2010, GSS 2012). This monitoring allows us greater scope to examine recent experiences of racism by ethnicity over time, alongside links between racism and health, with improved precision for specific ethnic groupings. Specifically this study aimed to: examine the prevalence of reported experience of racial discrimination (in the last 12 months), including changes over time, using multiple data sources (from the New Zealand General Social Surveys (GSS) and New Zealand Health Surveys (NZHS)); and examine the relationship between self-reported experience of racial discrimination (in the last 12 months) and measures of health and wellbeing, for both the overall adult population and stratified by major ethnic groupings.

## Methods

### Study design

This study undertakes a comprehensive analysis of secondary data from multiple national surveys of New Zealand adults, including the New Zealand Health Survey (2002/03, 2006/07, 2011/12) and the General Social Survey (2008, 2010, 2012). Both survey types allow for the calculation of nationally representative data through appropriate analysis incorporating the complex survey design (see details below). The surveys are repeated cross-sectional surveys.

The study followed Statistics New Zealand processes for accessing microdata [38]. Data were provided as confidentialised unit record files (CURFs) for all surveys. More detailed data on racism in particular settings were analysed in the Statistics New Zealand Data Lab for the GSS [39]. The study was approved by University of Otago Human Ethics Committee (D14/308).

### The surveys

#### The New Zealand Health Survey

The New Zealand Health Survey (NZHS) provides information on self-reported health status and chronic conditions, health risk and protective factors, experience and utilisation of health care and sociodemographic factors [40]. The NZHS has moved from a periodic survey (up until 2007) to a continuous survey (from 2011/12) with additional survey content rotated annually [40]. The three surveys selected for analysis (2002/03; 2006/07; 2011/12) are those that include questions on participant experiences of racial discrimination and were available at the beginning of data analysis.

The NZHS used multistage, probability-proportional-to-size (PPS) sampling methods to select participants from people who usually reside in New Zealand (usually resident) and were aged 15+ years (for the adult survey) [41]. In 2002/03 and 2006/07 this was restricted to residents in permanent, private dwellings and excluded those in institutions [41, 42]. In 2011/12 this was expanded to include people in non-private accommodation, such as aged-care facilities and student accommodation [40]. An area-based sampling frame used meshblocks (small areas of approximately 90 people) as primary sampling units, followed by random selection of households and individuals [40]. All instances of the NZHS employed methods to increase sampling of Māori, Pacific and Asian participants [40–42]. Computer assisted personal interviews (CAPI) were undertaken with participants by trained interviewers [40]. All results are weighted to account for survey design (including over-sampling) and non-response to provide representative results for the New Zealand adult population [40].

Table 1 summarises the NZHS instances used in this study with details on data collection period, participant numbers and response rates. Full details on each survey’s methods can be found elsewhere [40–42].

**Table 1:** Summary of surveys.

Survey	Participant age range	Data collection period	Number of adult participants	Response rate	Source
NZHS 02/03	15+	Aug 2002–Jan 2004	12,500	72%	[42]
NZHS 06/07	15+	Oct 2006–Nov 2007	12,488	68%	[41]
NZHS 11/12	15+	Jul 2011–June 2012	12,370	79%	[40]
GSS 2008	15+	Apr 2008–Mar 2009	8,721	83%	[43]
GSS 2010	15+	Apr 2010–Mar 2011	8,550	81%	[44]
GSS 2012	15+	Apr 2012–Mar 2013	8,462	78%	[45]

#### The General Social Survey

The GSS collects data biennially on adults aged 15 years and over who usually reside in New Zealand in private dwellings and do not reside in insitutions [43]. It provides nationally representative data on a range of social and economic indicators including life satisfaction, health, knowledge and skills, work, living standards, housing, physical environment, safety and security, support, social connectedness, leisure and recreation, culture and identity, and human rights [46].

The GSS has a three-stage sampling process. Primary sampling units (PSU) are selected from the Statistics New Zealand Household Survey Frame (HSF) based on NZ Census data. Individual dwellings are selected randomly within these PSUs, followed by random selection of eligible individuals within these dwellings [47]. The GSS is designed to produce nationally representative results. Data collection is via CAPI interviews [47]. Unlike the NZHS, the GSS does not employ methods to increase sampling of particular ethnic groups.

The first GSS was conducted in 2008. Table 1 summarises the three GSS instances used in this study with details on data collection period, participant numbers and response rates. The three surveys selected for this project were those available at the beginning of data analysis. Full details on the methods for each survey can be found elsewhere [43–45].

### Key variables

To enable comparisons across time periods and combination of results across survey instances, we prioritised selection of consistent variables (and categorisation of variables) across all surveys.

#### Racial discrimination

Both surveys (NZHS, GSS) provide data on participant experiences of racial discrimination, asked in either a one-step or two-step questioning process [48].

The NZHS uses a one-step process and asks directly about experience of racial/ethnic discrimination in New Zealand in five situations: physical and verbal attack, and unfair treatment because of ethnicity in health, housing and work situations. For example, “Have you ever been treated unfairly at work or been refused a job *because of your ethnicity* in New Zealand?”, with response options: Yes, within the past 12 months; Yes, more than 12 months ago; No; Don’t know; Refused [49]. Items were grouped for analysis into any experience of racial discrimination in the last 12 months if participants responded yes to any of the five items compared with no experience of racial discrimination in the last 12 months. Participant responses coded as “Don’t know” or “Refused” were excluded from analyses.

The GSS uses a two-step process whereby participants are initially asked about any experience of discrimination in the last 12 months (response options: Yes, No, Don’t know, Refused), with a follow-up question on the attribution of discrimination e.g. due to skin colour, nationality, age, gender etc. Experience of racial discrimination for this study was categorised based on discrimination reported as being due to ‘skin colour’, and/or ‘nationality, race or ethnic group’ in any setting. This is in line with NZ legislative and policy definitions of racism and racial discrimination whereby ‘colour’, ‘race’ and ‘ethnic or national origins’ are prohibited grounds for discrimination (Human Rights Act 1993). For analysis over time, and in examining associations between racism and health, any experience of racism in the last 12 months was used in analyses compared with no experience of racism in the last 12 months. The number of times participants experienced discrimination overall was also asked but could not be analysed for racial discrimination specifically [50].

The GSS also asks about the settings where discrimination occurred. In this study, settings where people experienced racial discrimination were examined. Some settings options were grouped where they were examining similar areas. These groups were ‘in public’ (grouping the response options: on the street or in a public place of any kind; using transport of any kind; getting service where buying something), in work (at work or while working; applying for or keeping a job or position), in justice (dealing with the police; dealing with the courts). Other settings were: at home (single response option); school (getting into a school or other place of learning, or being treated fairly there); joining an association or club of any kind (single response option); housing (applying for or keeping a flat or housing of any kind); dealing with other government officials (single response option); health (dealing with people involved in health care); and Other (with no further detail available in the source data).

The two approaches for how the discrimination questions were asked across the NZHS and GSS can be found in S1 Table.

#### Ethnicity

The NZHS and GSS use the standard NZ Population Census ethnicity question that allows people to self-identify their ethnic group or groups. The question provides a number of ethnic group response options, along with an “Other” category accompanied by a free-text field where people can write in their ethnicity if it is not captured by the response options. NZ uses a hierarchical classification system to classify responses to the ethnicity question from Level 1 (broad aggregate groupings) to Level 4 (detailed ethnic groups). For prevalences of experience of racial discrimination, ethnicity was categorised as total Māori, total Pacific, total Asian compared to a mutually exclusive European/Other group (see [51] for information on the specific ethnic groups included within these categories). Total response ethnicity refers to the way in which people who report more than one ethnicity are categorised for analysis, by which they are counted in each of the major ethnic groupings with which they identify [51]. For multiple regression analyses, ethnicity was grouped into four mutually exclusive categories using prioritisation [51] in the following order: Māori, Pacific, Asian and European/Other. Several of the CURF datasets (NZHS 2011/12, and GSS 2008) did not allow for disaggregation of people who identified as European or ‘Other’. To maintain consistency we used the European/Other grouping for all datasets and analyses. Based on datasets where disaggregation of European and Other ethnic groups could be undertaken (NZHS 2002/03, 2006/07, GSS 2010, 2012) it appears that this group is predominantly European (‘Other’ ethnic groups made up 1–4% of the European/Other group).

#### Health and wellbeing

Health variables common to both survey types were included to examine associations between racial discrimination and health. These included, self-rated general health (poor/fair vs. good/very good/excellent), and SF-12 mental and physical health component summary scores (analysed as continuous variables) [52]. Analysis of SF-12 data excludes the 2002/03 NZHS as this used version 1 of the SF-12. To include a broader measure of wellbeing, life satisfaction was also analysed for the GSS data only (responses grouped into dissatisfied/very dissatisfied vs. no feeling either way/satisfied/very satisfied).

#### Other covariates

Other variables considered in multivariable models include: age (15–24, 25–34, 35–44, 45–64, 65–74, 75+ years); gender (male, female); and nativity (born in NZ vs. born overseas). Socioeconomic variables included an area-based measure (New Zealand Index of Deprivation (NZDep): quintiles from 1 (least deprived) to 5 (most deprived) [53], and an individual measure (education qualification: no secondary qualification, secondary qualification or higher).

### Data analysis

All analyses accounted for the complex survey nature of the dataset, and adjusted for both inverse sample weighting, stratification of the sampling frame, and clustering by PSU (see survey sampling method above.) Analyses of the individual survey instances were conducted in SAS 9.4 (SAS Institute, Cary, NC) using the SURVEYFREQ, SURVEYLOGISTIC, and SURVEYREG procedures. Random effects meta-analysis to combine regression estimates across the six NZHS/GSS survey instances were conducted in R 3.2 (R Institute, Vienna, Austria) using the meta package [54].

Reported experience of any racial discrimination (in the last 12 months) by major ethnic grouping was analysed for each survey. Prevalences of racial discrimination by setting were analysed for the GSS 2008, 2010, and 2012 in the Statistics New Zealand data lab and are subject to data lab confidentiality rules related to outputs [39]. Trends over time by ethnic grouping were visualised using a trend line weighted to the inverse variance of the prevalence estimates (see e.g. [55])

Multiple regression analysis was undertaken in each individual survey instance to examine the sociodemographic patterning of reported experience of racial discrimination in the last 12 months (compared with no reported experience of racial discrimination in the last 12 months), across all surveys. Standard random-effects meta-analysis methods [54, 56, 57] were used to combine estimated effect sizes across the six unique survey instances. Sociodemographic variables included: age, gender, education qualification, NZDep quintiles, ethnicity and nativity. Nativity had an effect on experience of racial discrimination for some ethnic groups, and therefore the levels of the ethnic/nativity group variables were combined and analysed with NZ-born European/Other as the reference group.

Multiple regression analyses were used to examine associations between experience of racial discrimination in the last 12 months (compared with no reported experience of racial discrimination in the last 12 months) and health and wellbeing measures for each survey. These analyses were conducted for the total adult samples, and also stratified by ethnicity (to determine the impact of racism within each ethnic grouping). Logistic regression was used for binary health variables (life satisfaction, self-rated health) and linear regression for contiuous measures (SF-12 mental and physical scores). Logistic regression analyses present the odds ratios (ORs) for poor health outcomes comparing those reporting experience of racism in the last 12 months to those not i.e. a positive OR indicates a relationship between experience of racism and poor health or dissatisfaction. Linear regression models present the mean difference in SF-12 scores for those reporting experience of racism in the last 12 months and those not i.e. a negative results indicates an association between experience of racism and negative health.

Meta-analysis techniques using random effects models were used to combine effect sizes. Models presented in the main text of this manuscript were adjusted for age, gender, ethnicity, nativity, NZDep and education qualification; and adjusted for age, gender, nativity, NZDep and education qualification in ethnicity-stratified models. Estimates from the unadjusted models are presented in S2 Fig and S5 Table respectively. Formal hypothesis tests are reported for whether the impact of recent racism on each health outcome differs between ethnic groupings (formally considered as a sub-group comparison of the pooled effect sizes from the ethnicity-stratified estimates, using Cochran’s Q test) [56, 57].

## Results

Table 2 shows the total number of participants and their sociodemographic characteristics for each survey analysed. The majority of participants in the Asian (90–94%) and Pacific (57–67%) ethnic groups were born overseas, while nearly all participants who identified as Māori were born in New Zealand (97–99%).

**Table 2:** Sociodemographic characteristics of participants by survey.

Variable (level)	GSS 2008		GSS 2010		GSS 2012		NZHS 02/03		NZHS 06/07		NZHS 11/12	
	Freq	Percent (95% CI)	Freq	Percent (95% CI)	Freq	Percent (95% CI)	Freq	Percent (95% CI)	Freq	Percent (95% CI)	Freq	Percent (95% CI)
Total	8721	100	8550	100	8462	100	12529	100	12488	100	12596	100
Ethnicity (nativity*)												
Māori	972	12.5 (12.4, 12.6)	947	12.6 (12.6, 12.6)	1114	12.7 (12.7, 12.8)	4120	10.9 (10.9, 10.9)	3160	11.4 (11.4, 11.4)	2586	12.6 (12.5, 12.7)
* NZ born*	*957*	*98*.*3 (97*.*3*, *99*.*3)*	*935*	*98*.*4 (97*.*4*, *99*.*5)*	*1091*	*97*.*6 (96*.*6*, *98*.*7)*	*4087*	*98*.*8 (98*.*1*, *99*.*6)*	*3130*	*98*.*9 (98*.*4*, *99*.*4)*	*2556*	*98*.*3 (97*.*4*, *99*.*1)*
* Overseas born*	*15*	*1*.*7 (0*.*7*, *2*.*7)*	*12*	*1*.*6 (0*.*5*, *2*.*6)*	*23*	*2*.*4 (1*.*3*, *3*.*4)*	*33*	*1*.*2 (0*.*4*, *1*.*9)*	*30*	*1*.*1 (0*.*6*, *1*.*6)*	*30*	*1*.*7 (0*.*9*, *2*.*6)*
Pacific	353	5.8 (4.8, 6.9)	263	4.2 (3.4, 5.0)	378	4.9 (4.4, 5.5)	908	4.4 (4.3, 4.5)	918	5.0 (4.9, 5.1)	849	5.4 (5.2, 5.5)
* NZ born*	*144*	*42*.*9 (37*.*1*, *48*.*7)*	*100*	*35*.*9 (28*.*2*, *43*.*6)*	*146*	*39*.*9 (34*.*0*, *45*.*8)*	*258*	*32*.*7 (28*.*3*, *37*.*1)*	*352*	*41*.*5 (37*.*4*, *45*.*6)*	*290*	*40*.*2 (34*.*9*, *45*.*6)*
* Overseas born*	*209*	*57*.*1 (51*.*3*, *62*.*9)*	*163*	*64*.*1 (56*.*4*, *71*.*8)*	*232*	*60*.*1 (54*.*2*, *66*.*0)*	*649*	*67*.*3 (62*.*9*, *71*.*7)*	*565*	*58*.*5 (54*.*4*, *62*.*6)*	*558*	*59*.*8 (54*.*4*, *65*.*1)*
Asian	520	8.3 (7.4, 9.1)	560	9.7 (8.7, 10.8)	618	11.0 (9.7, 12.2)	1172	6.0 (6.0, 6.0)	1463	8.8 (8.7, 8.8)	866	10.3 (10.1, 10.4)
* NZ born*	*36*	*5*.*8 (3*.*7*, *7*.*8)*	*38*	*6*.*0 (3*.*7*, *8*.*3)*	*49*	*8*.*9 (5*.*9*, *11*.*8)*	*61*	*5*.*9 (3*.*7*, *8*.*1)*	*109*	*7*.*9 (6*.*1*, *9*.*6)*	*71*	*10*.*0 (6*.*8*, *13*.*2)*
* Overseas born*	*484*	*94*.*2 (92*.*2*, *96*.*3)*	*522*	*94*.*0 (91*.*7*, *96*.*3)*	*569*	*91*.*1 (88*.*2*, *94*.*1)*	*1111*	*94*.*1 (91*.*9*, *96*.*3)*	*1354*	*92*.*1 (90*.*4*, *93*.*9)*	*794*	*90*.*0 (86*.*8*, *93*.*2)*
European & Other	6865	73.4 (72.2, 74.6)	6779	73.4 (72.1, 74.7)	6348	71.3 (70.0, 72.6)	6329	78.7 (78.6, 78.9)	6947	74.9 (74.8, 75.0)	8295	71.8 (71.6, 72.0)
* NZ born*	*5587*	*81*.*1 (80*.*1*, *82*.*2)*	*5462*	*79*.*1 (77*.*8*, *80*.*4)*	*5158*	*79*.*5 (78*.*2*, *80*.*9)*	*5170*	*82*.*4 (81*.*0*, *83*.*8)*	*5508*	*79*.*1 (78*.*0*, *80*.*3)*	*6760*	*79*.*7 (78*.*1*, *81*.*3)*
* Overseas born*	*1278*	*18*.*9 (17*.*8*, *19*.*9)*	*1317*	*20*.*9 (19*.*6*, *22*.*2)*	*1188*	*20*.*5 (19*.*1*, *21*.*8)*	*1159*	*17*.*6 (16*.*2*, *19*.*0)*	*1438*	*20*.*9 (19*.*7*, *22*.*0)*	*1531*	*20*.*3 (18*.*7*, *21*.*9)*
Missing ethnicity	11		1		4		0		0		0	
Missing nativity	0		0		2		1		2		6	
**Age group**												
15–24	958	18.2 (18.2, 18.2)	912	18.3 (18.3, 18.3)	935	18.0 (18.0, 18.0)	1566	17.3 (17.3, 17.3)	1663	17.7 (17.7, 17.7)	1488	18.2 (18.2, 18.2)
25–34	1277	16.2 (16.2, 16.2)	1146	16.2 (16.2, 16.2)	1217	16.5 (16.5, 16.5)	2344	18.3 (18.3, 18.3)	2080	16.3 (16.3, 16.3)	1958	16.2 (16.2, 16.2)
35–44	1690	18.7 (18.7, 18.7)	1652	17.7 (17.7, 17.8)	1467	16.9 (16.8, 16.9)	2695	20.5 (20.5, 20.6)	2577	19.5 (19.5, 19.5)	2372	16.9 (16.9, 16.9)
45–54	1547	17.9 (17.9, 17.9)	1502	17.9 (17.9, 17.9)	1519	17.7 (17.6, 17.7)	2040	17.3 (17.3, 17.3)	2079	17.8 (17.8, 17.8)	2124	17.5 (17.5, 17.5)
55–64	1317	13.7 (13.7, 13.7)	1359	14.1 (14.1, 14.1)	1379	14.3 (14.3, 14.3)	1678	11.8 (11.8, 11.8)	1729	13.4 (13.4, 13.4)	1960	14.1 (14.1, 14.1)
65–74	1021	8.7 (8.7, 8.7)	1033	9.1 (9.1, 9.1)	1074	9.8 (9.8, 9.8)	1236	8.0 (8.0, 8.0)	1304	8.6 (8.6, 8.6)	1415	9.5 (9.5, 9.5)
75+	911	6.6 (6.6, 6.6)	946	6.7 (6.7, 6.7)	871	6.9 (6.9, 6.9)	970	6.8 (6.8, 6.8)	1056	6.7 (6.7, 6.7)	1279	7.5 (7.5, 7.5)
**Gender**												
F	4800	51.5 (51.5, 51.6)	4773	51.4 (51.4, 51.4)	4749	51.3 (51.3, 51.3)	7658	51.9 (51.9, 51.9)	7215	52.0 (52.0, 52.0)	7488	51.4 (51.4, 51.4)
M	3921	48.5 (48.4, 48.5)	3777	48.6 (48.6, 48.6)	3713	48.7 (48.7, 48.7)	4871	48.1 (48.1, 48.1)	5273	48.0 (48.0, 48.0)	5108	48.6 (48.6, 48.6)
**Education level**												
No secondary qualification	2296	23.5 (22.1, 24.8)	2077	21.4 (20.1, 22.7)	2041	20.6 (19.4, 21.8)	4948	30.9 (29.6, 32.2)	4007	26.7 (25.8, 27.7)	3872	25.5 (24.2, 26.8)
Secondary qualification	6405	76.5 (75.2, 77.9)	6459	78.6 (77.3, 79.9)	6412	79.4 (78.2, 80.6)	7574	69.1 (67.8, 70.4)	8456	73.3 (72.3, 74.2)	8643	74.5 (73.2, 75.8)
Missing	20		14		9		7		25		81	
**NZDep Quintile**												
1 (least deprived)	1561	22.1 (19.4, 24.7)	1518	21.4 (18.8, 24.0)	1418	21.9 (19.1, 24.6)	1705	18.5 (18.5, 18.5)	2011	21.6 (19.0, 24.2)	1942	20.6 (20.1, 21.0)
2	1830	20.5 (17.9, 23.2)	1784	19.3 (16.7, 21.8)	1494	21.5 (18.5, 24.6)	1573	19.4 (19.4, 19.5)	2122	19.5 (17.2, 21.8)	1962	20.4 (20.1, 20.8)
3	1890	20.1 (17.3, 22.9)	1874	21.6 (18.4, 24.7)	1534	19.1 (16.7, 21.6)	1854	20.5 (20.5, 20.5)	2501	20.7 (18.5, 22.8)	2514	20.2 (19.9, 20.6)
4	1924	19.4 (17.3, 21.6)	1898	19.8 (17.6, 22.0)	2094	20.7 (18.3, 23.1)	2389	21.6 (21.6, 21.7)	2731	20.5 (18.4, 22.6)	2708	19.9 (19.5, 20.3)
5 (most deprived)	1488	17.9 (15.8, 20.0)	1449	17.9 (15.7, 20.1)	1922	16.8 (15.0, 18.5)	4979	19.9 (19.8, 20.0)	3123	17.7 (15.5, 19.9)	3458	18.8 (18.4, 19.3)
Missing	28		27		0		29		0		12	

Unweighted frequencies and weighted percentages.

*Nativity is presented as proportion born in New Zealand (NZ) or overseas within each prioritised ethnicity group.

**Fig 1:**
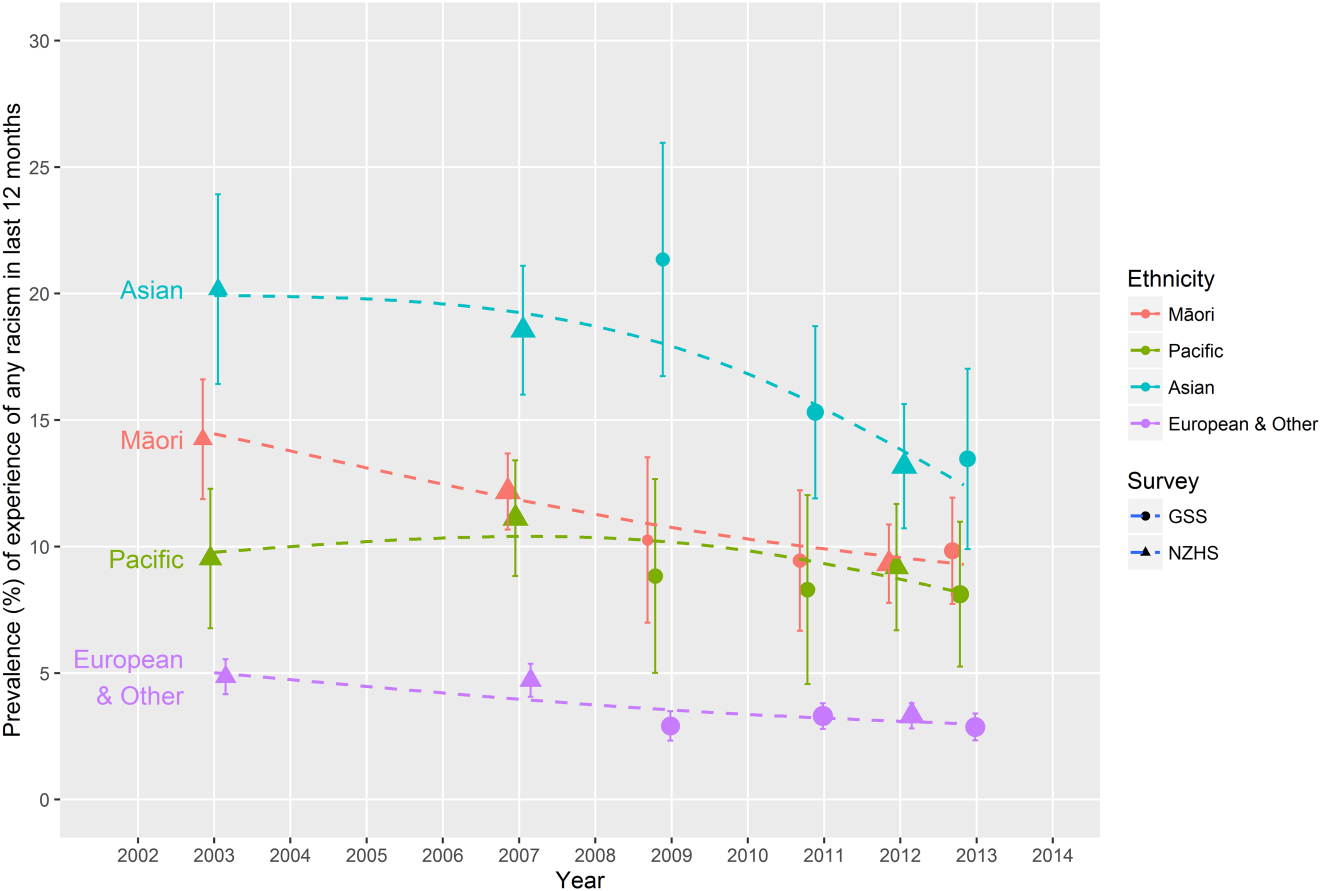
Prevalence of experience of racism in the last 12 months over time by ethnicity and survey, NZHS 2002/03, 2006/07, 2011/12, GSS 2008, 2010, 2012.

Fig 1 shows reported experience of racial discrimination in the last 12 months organised by major ethnic grouping over the study period (from the 2002/03 NZHS to the GSS 2012). There was some variation in the prevalence estimates over time and between survey instances, particularly for the non-European ethnic groups. Asian participants reported the highest experience of racism (around 13–15% prevalence over the three most recent surveys), followed by Māori and Pacific (8–10% in three most recent surveys). Europeans (European/Other) reported significantly lower experience of racism than the other groupings (around 4% over last three surveys). With the exception of Pacific peoples, reporting of recent racial discrimination appears to be declining over the time period examined. Prevalence estimates and confidence intervals are presented in S2 Table.

Table 3 shows the pooled estimates from meta-analysis for patterning of reported experience of racial discrimination in the last 12 months by sociodemographic variables, pooled across all six survey instances. Recent experience of racial discrimination was higher among men compared to women, and among younger age groups. Reported recent experience of racism decreased with age, with lower rates reported among the oldest two age categories compared to the youngest. Reporting recent experience of racism also increased with higher levels of area deprivation. Māori, Asian and Pacific participants all reported significantly higher experience of recent racial discrimination than participants in the NZ-born European/Other group, independent of whether they were born in New Zealand or not. Nativity was also important among Asian and European/Other ethnic groups, with those born overseas reporting higher rates of racism compared to their NZ-born counterparts in the same ethnic grouping. Overseas-born Asian participants in particular reported odds of 5.53 that of NZ-born European/Other participants, which was about twice as high as for NZ-born Asian respondents. Results by individual survey are available in S3 Table.

**Table 3:** Association of sociodemographic factors and experience of racism (last 12 months), from meta-analysis of multivariable logistic regression across all six surveys (NZHS and GSS).

Variable	Level		Recent experience of racism
			Odds Ratio (95% CI)
Ethnicity/ Nativity	European/Other	NZ born	1 (ref)
		Overseas born	2.16 (1.70, 2.74)
	Māori	NZ born	3.08 (2.45, 3.86)
		Overseas born*	*Not reported*
	Pacific	NZ born	1.96 (1.44, 2.65)
		Overseas born	2.23 (1.62, 3.07)
	Asian	NZ born	2.63 (1.54, 4.49)
		Overseas born	5.53 (4.52, 6.78)
Age group		15–24	1 (ref)
		25–34	0.81 (0.67, 0.97)
		35–44	0.86 (0.70, 1.07)
		45–54	0.83 (0.60, 1.14)
		55–64	0.60 (0.45, 0.78)
		65–74	0.28 (0.19, 0.43)
		75+	0.13 (0.09, 0.19)
Gender		Male	1.19 (1.09, 1.31)
		Female	1 (ref)
Education	No secondary qualification		1.01 (0.90, 1.14)
	Secondary qualification		1 (ref)
NZDep Quintile		1	1 (ref)
		2	1.19 (0.96, 1.48)
		3	1.43 (1.21, 1.69)
		4	1.55 (1.28, 1.87)
		5	1.64 (1.26, 2.15)

Odds ratios are from random effects meta-analysis of all six surveys: NZHS 2002/03, 2006/07, 2011/12, GSS 2008, 2010, 2012.

Ethnicity is prioritised in the following order: Māori, Pacific, Asian, European/Other.

*Māori overseas born are included in the model but data is not shown because of small numbers.

**Fig 2:**
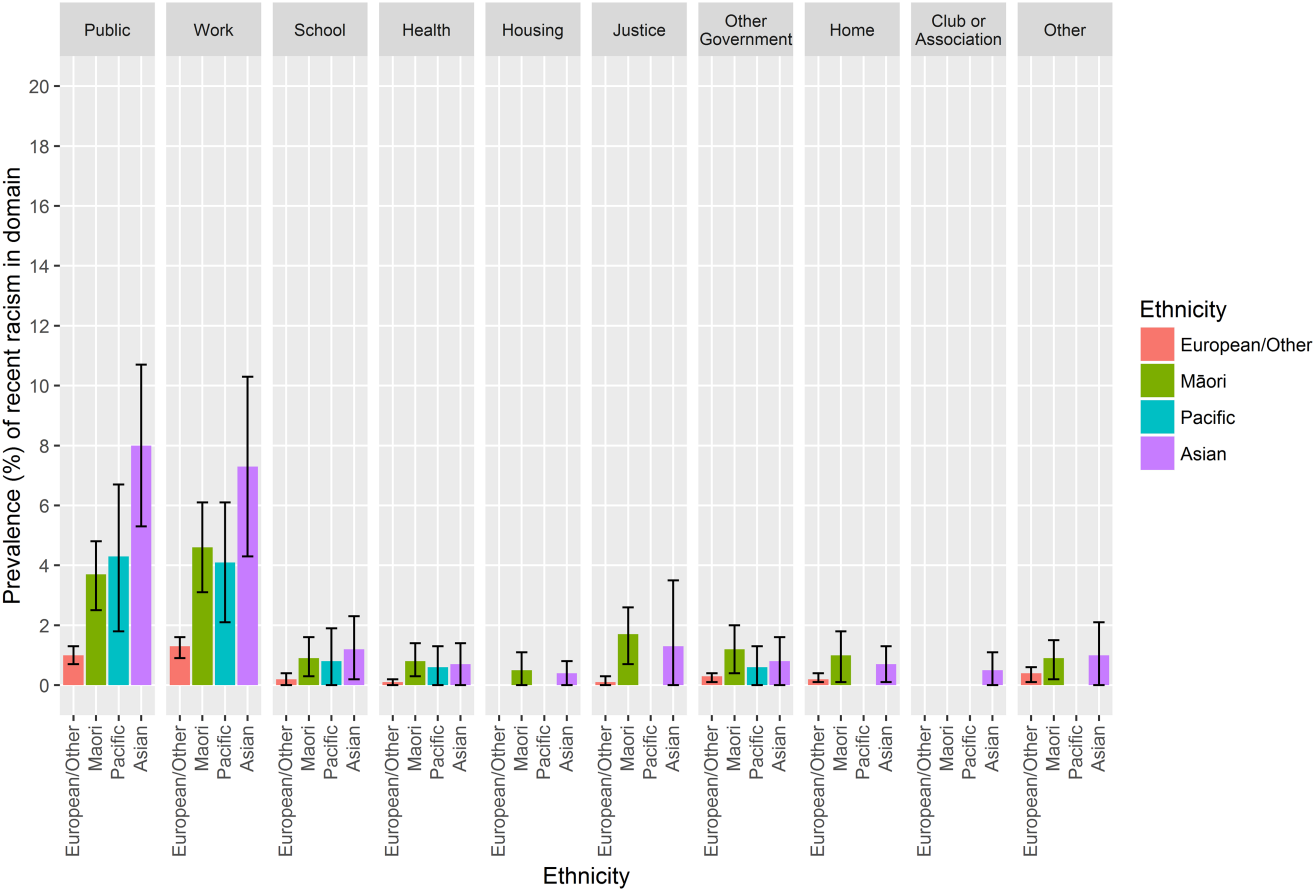
Prevalence (%) of experience of racism by setting and ethnicity, GSS 2012. Figure note: Data is from the Statistics New Zealand data lab.

The GSS enabled examination of the settings in which participants reported experiencing racism. The two most common such settings were public settings (including on the street, using transport and service when buying something) and work settings (including at work or when working, and applying for or keeping a job or position) (Fig 2, showing reporting of racism by setting for the GSS 2012). For these settings, prevalences were highest for Asian participants in all survey instances, followed by Māori and Pacific groups, with lowest rates among the European/Other group. More detailed prevalence data and confidence intervals are appended for GSS 2008, 2010, and 2012 (S4 Table and S1 Fig).

**Fig 3:**
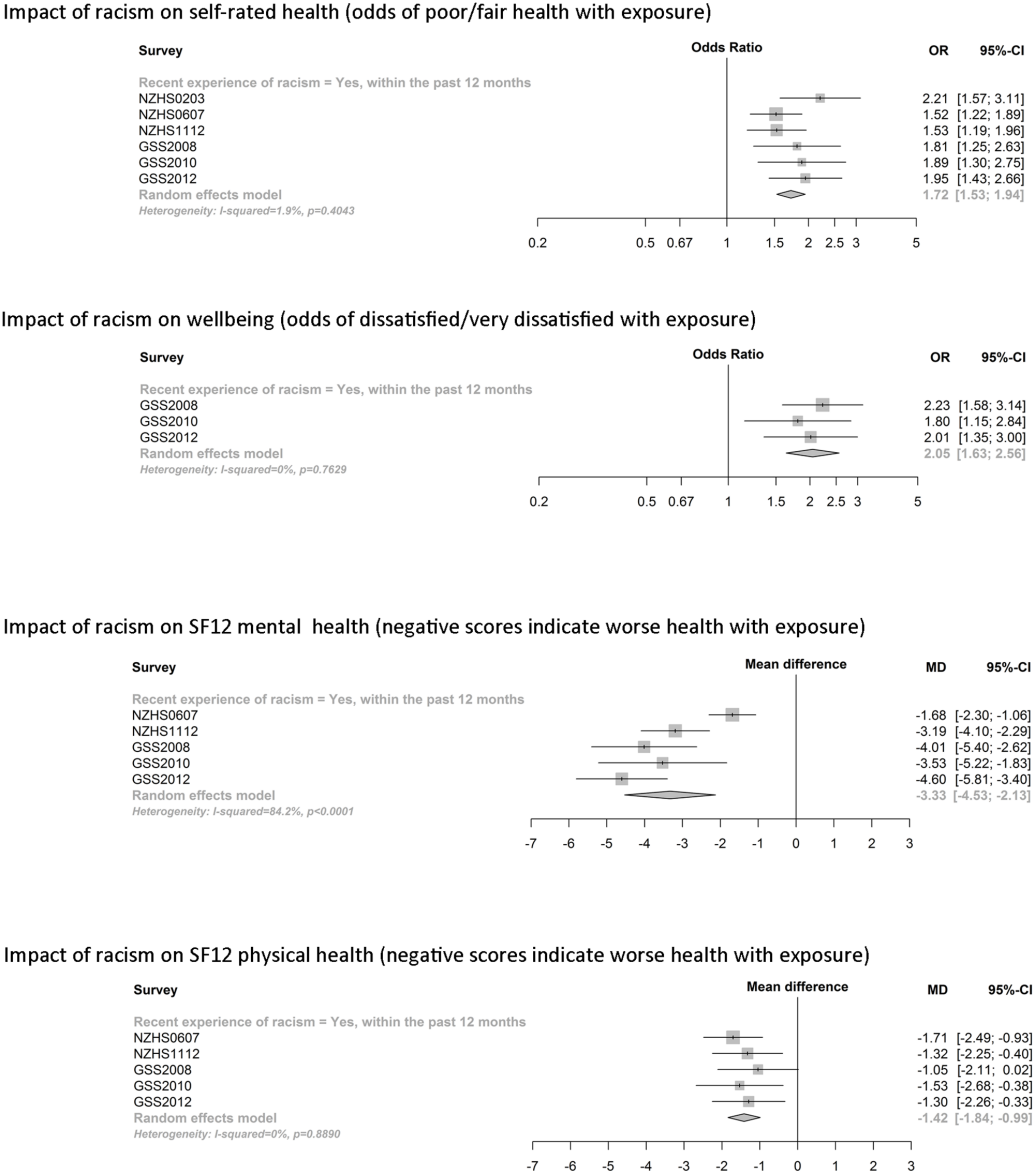
Association between experience of racial discrimination and health and wellbeing measures, by survey and combined in meta-analysis. Adjusted for age, gender, ethnicity, nativity, education qualification, NZDep.

Fig 3 shows forest plots demonstrating the relationship between any experience of racism in the last 12 months and each health and wellbeing outcome, adjusted for age, gender, ethnicity, nativity, NZDep and education. These are reported for each survey (squares and horizontal lines giving the point estimate and 95% CI for each survey instance) and the pooled effect size from meta-analysis (diamond at bottom of each health outcome figure). All surveys showed an association between recent experience of racism and each of the negative health and wellbeing measures examined. The pooled results from the meta-analysis showed that racism is strongly and significantly linked to negative measures of self-rated health, mental health (SF-12), physical health (SF-12) and general wellbeing (life satisfaction). Corresponding results from unadjusted models can be found in S2 Fig.

The final analyses further examined the relationship between racism and health/wellbeing measures stratified by respondent ethnicity. Table 4 presents pooled effect sizes for each ethnic grouping from the meta-analyses, adjusted for age, gender, nativity, NZDep and education. Effect sizes for the individual survey analyses are in S3, S4, S5 and S6 Figs. where it can be seen that confidence intervals were wide for any single survey instance, particularly for the smaller ethnic groupings (Pacific and Asian). Experience of racism in the last 12 months was generally associated with negative health and wellbeing measures for all ethnic groups, with a few variations seen between ethnic groupings. Recent experience of racism was associated with negative self-rated health for each ethnic grouping, with no major differences in the estimated impact, as can be seen by comparing point estimates and confidence intervals across stratified ethnic groupings (test for sub-group differences: Cochran’s Q (3df) = 2.71, p = 0.438). Analysis of the life satisfaction outcome could only be undertaken using GSS data and was most affected by small numbers in ethnically stratified results. Therefore, confidence intervals from the meta-analysis were wide and were not significant for Māori and Pacific groups. However, point estimates for all ethnic groups demonstrated an association between racism and lower life satisfaction, with no apparent differences in the strength of association by ethnicity (test for sub-group differences: Q (3df) = 3.76, p = 0.288). Similarly, for the SF-12 mental health scores, experience of racism remained associated with subsequently poorer mental health for all ethnic groupings, with no obvious differences by ethnicity grouping (test for sub-group differences: Q (3df) = 5.40, p = 0.145). The impact of racism on SF-12 physical health scores appeared to differ by ethnicity, with no evidence for a strong association with poorer physical health for Asian participants (mean difference close to 0) but a more substantial difference for other ethnic groups (test for sub-group differences: Q (3df) = 28.20, p < 0.001). Unadjusted findings by individual survey are available in S5 Table.

**Table 4:** Meta-analysis of surveys showing association between experience of racial discrimination (last 12 months) and health and wellbeing measures, stratified by ethnicity and adjusted for age, gender, nativity, NZDep, education qualification.

Health and wellbeing variables	Ethnicity	Impact of racism on health outcome
		Adjusted Odds Ratio (95% CI)
Poor/fair self-rated health^a^	European/Other	1.98 (1.55, 2.51)
	Māori	1.89 (1.53, 2.32)
	Pacific	1.73 (1.16, 2.57)
	Asian	1.39 (0.96, 2.01)
Dissatisfied/very dissatisfied life satisfaction^b^	European/Other	2.33 (1.73, 3.14)
	Māori	1.39 (0.87, 2.20)
	Pacific	1.84 (0.70, 4.82)
	Asian	2.41 (1.31, 4.44)
		Mean difference (95% CI)
SF-12 mental health^c^	European/Other	-2.94 (-4.35, -1.54)
	Māori	-4.10 (-5.19, -3.01)
	Pacific	-3.56 (-5.19, -1.93)
	Asian	-2.40 (-3.40, -1.39)
SF-12 physical health^c^	European/Other	-2.41 (-3.11, -1.72)
	Māori	-0.93 (-2.06, 0.20)
	Pacific	-2.23 (-3.73, -0.72)
	Asian	0.18 (-0.53, 0.89)

Note: Odds ratios are from random effects meta-analysis of surveys as noted for each outcome. Ethnicity is prioritised in the following order: Māori, Pacific, Asian, European/Other.

^a^(NZHS 2002/03, 2006/07, 2011/12, GSS 2008, 2010, 2012);

^b^(GSS 2008, 2010, 2012);

^c^(NZHS 2006/07, 2011/12, GSS 2008, 2010, 2012).

## Discussion

This study focuses on New Zealand adults’ reporting of recent experience of racism (in the last 12 months) utilising data from multiple repeated cross-sectional national surveys over a ten-year period (NZHS 2002/03 to GSS 2012). Recent experience of racism was most commonly reported by Asian participants followed by Māori and Pacific peoples, with the largely European group (European/Other) reporting the lowest experience of racism. Among Asian participants, reported experience of racism was higher for those born overseas compared to those born in New Zealand. Encouragingly, reported recent experience of racism appears to be declining for most of these groups over the time period examined.

The results also demonstrated a consistent association of recent experience of racism with negative measures of health and wellbeing, including indicators of mental health (SF-12 mental health summary score), physical health (SF-12 physical health summary score and self-rated general health) and overall life satisfaction. While the impact of experience of racism appeared similar when analyses were stratified by ethnicity (with the exception of the SF-12 physical health), it is important to note that the burden of higher prevalence of racism for Māori, Pacific, and Asian groups means that the population-level impact of racism disproportionately affects these groups.

As expected the prevalence of reported racism in the last 12 months was lower than experience ‘ever’, as can be seen by comparing data from the NZHS in this study with previously published data from 2002/03 and 2006/07 [16, 17]. The finding of associations between recent experience of racism and negative health and wellbeing measures is consistent with other New Zealand evidence. Reported experience of racism ‘ever’ in the NZHS has been significantly associated with poorer self-rated health, and measures of physical and mental health [16, 17]. Reported experience of racism has also been associated with lower life satisfaction in longitudinal NZ research [24]. The findings from our study are also consistent with the body of international evidence demonstrating associations between experience of racism and negative mental health, physical health and general health [3, 4] and replicate the finding that there is a stronger association with negative mental health than with physical health [3]. Evidence also suggests that recent experience of racism may be more strongly associated to mental health and more weakly related to life satisfaction measures [3]. However, our study is limited in assessing this, in part as the GSS surveys only ask about recent experience of racism. Differences in study design and study variables (both mental health and racial discrimination) mean findings are not directly comparable with studies that have examined associations with longer-term experiences of racism [16, 17, 24].

A major strength of this study is the documentation of recent experience of racism from multiple national studies at six time points to examine trends in self-reported experience of racism over time. Previous analyses of NZHS and GSS data have documented prevalence estimates of experience of racism and relationships with health for individual studies [13, 16, 17, 20, 58, 59]. However, for NZHS data, this is usually focussed on lifetime experience of racism (16, 22) and for GSS data, focuses on discrimination more broadly [20, 60] and has not examined trends over time [20]. The apparent reduction in reporting of racial discrimination over the time period for Māori, Asian and European/Other is encouraging, although the specific reasons for this cannot be determined. It is important when examining reported experiences of racial discrimination over time to consider the potential for changes in the way that racism may be expressed, understood, recognised and reported. In our study, we see a decreasing prevalence of reporting racism with increasing age. This may reflect differences in exposure to racism, with earlier research showing that for the NZHS this relationship is driven by higher prevalences of the personal attack variables in younger age groups [16]. However, this age relationship is still seen in ‘ever’ reporting of racism, suggesting we may also need to consider birth cohort influences.

Another strength of this study is the examination of how nativity influences experience of racial discrimination in New Zealand. The majority of participants in the Asian and Pacific ethnic groupings were born outside of New Zealand. Our findings showed that experience of racial discrimination for Asian and European/Other people born outside of New Zealand was higher than those born in New Zealand. In the US, where the majority of literature on racism and health is situated, most studies that examine the effect of nativity have found that reporting of racism among people from minoritised racial/ethnic groups born outside of the US is lower than their US-born counterparts (e.g. for Black, Latino/a and Asian groups) [61]. However, this is not always the case, with the reverse sometimes seen and variation in the consistency of findings by racial/ethnic group [61]. We cannot determine (based on our study) why these differences might exist between our NZ population and the majority of US studies. It is important to note that ethnic groupings used in this study (with the exception of Māori) are composite categories, and include multiple ethnic groups that may not be directly comparable to similar US racial/ethnic categories with regards to their underlying composition and time since immigration into the respective country. Contextual factors with regards to the country of study may also influence differences in exposure, recognition and reporting of racism, as well as differences in methods to elicit experiences of racism. The inability to disaggregate the European/other ethnic grouping in this study is also a limitation with regard to understanding the role of nativity in exposure to racism for particular ethnic groups within this category as we cannot determine if the higher rate of racism by overseas-born people in this category is more likely to be non-European participants (though such participants are likely to make up a small proportion of this group, as noted in the methods).

This study was also able to present the most common settings that people experience racism from the GSS data. These were at work and in public, which is in line with the settings for experiences of discrimination more broadly [20]. It is important to understand where people experience racism as this has direct implications for interventions, particularly in terms of agency and responsibility for preventing such discrimination [62]. For example, in terms of workplace safety, employers in New Zealand are responsible for providing safe working environments, including non-discriminatory environments, under the Employment Relations Act 2000.

Another study strength is the use of meta-analysis to combine data over multiple survey instances for improved precision of estimates. We used meta-analysis methods [63, 64] to pool estimates across the six survey instances. This allows for direct visualisation of how effect sizes differ across individual surveys (e.g. see Fig 3) while also providing improved precision for the pooled estimate compared to considering results from the single survey instances independently. This assumes that the association between experience of racism and the health outcomes is reasonably consistent over time, following adjustment for confounders (so changes to confounder profiles by exposed/unexposed groups over time are accounted for as well). We considered this to be a reasonable assumption given the same target population for all survey instances (general NZ population) and the short overall period of the included surveys (over ten years). As can be seen in the forest plots, estimates for the impact of racism on health were generally consistent across survey instances.

As per any meta-analysis, there is no particular requirement that outcomes and exposures be measured in the exact same manner across studies [65]: an additional strength of this study is that the study outcomes were chosen to be identical across all survey instances, and the target population for the surveys is identical between the GSS and the NZHS (the adult NZ population). While the exact wording of the racism questions differed in the two main surveys, as described above, the underlying construct and timing of experience of racism are consistent enough that this represents a consistent definition of exposure that is suitable for meta-analysis. The use of random-effects models also allows potential variation in effect size (due to exact exposure definition) to be reflected in wider confidence intervals for the pooled estimates.

The relationship between reported racism and health has previously been examined using data from the NZHS for 2002/03 and 2006/07 data separately, although again this has largely used experience of racism ‘ever’ in a person’s life-time [16–18, 22], with these papers noting that the smaller numbers reporting racism in the last 12 months limited analytical power for individual surveys [17]. The use of meta-analysis was particularly useful in providing improved estimation of this association, with greater statistical precision. It has also allowed for improved estimates between recent experience of racism and health/wellbeing by ethnic grouping, another limitation noted in previous research [3, 16] and has allowed for the quantification of the extent to which racism affects health differently in particular ethnic groups [3]. In our study, we found that recent experience of racism was similarly associated with negative mental health, self-rated health and life satisfaction. However, no association with physical health was found among the Asian ethnic grouping. The relationship between experience of racism and physical health outcomes is less consistent than for mental health measures [5]. While there is a long history of Asian immigration to New Zealand [66], this finding may be influenced by the large proportion of people in the Asian category born outside of New Zealand and the potential influence of the "healthy immigrant” effect [8, 62]. It should be noted that this advantage has been shown to fade over time, and the relationship between racism and negative health has been reported to strengthen over time for Asian immigrants to the US [8]. Future studies focusing on the role of nativity in this question would be advised to consider time spent in New Zealand in analyses.

A number of limitations should be considered in the interpretation of findings. Our study is cross-sectional and therefore limited in terms of attributing causality. However, experience of racism has been linked to negative health and well-being outcomes in prospective studies, both in New Zealand [23, 24] and internationally [3].

In addition, our prevalence estimates for experience of racism may be underestimates, particularly for non-European groups. Firstly, questions asked about a limited number of settings and forms of racism (e.g. personal attack in the NZHS). Previous research has shown that Māori, Asian and Pacific groups are more likely to experience multiple forms of racial discrimination [16, 17]. Widening the range of settings and forms, and capturing frequency of exposure may improve measurement in future studies. We also note that under-reporting of racial discrimination may be higher for marginalised groups with reporting influenced by several factors including, difficulty recognising racism towards oneself (compared to one’s group), social desirability bias and reluctance to report experiences, explicit versus implicit cognition of racism, and the impact of internalised racism on recognition and reporting of racism [62, 67, 68, 69]. Under-reporting of exposure may in turn lead to underestimation of associations between racism and health or wellbeing. The measurement of experience of racial discrimination in the last 12 months also underestimates people’s longer-term experiences of racism and may contribute to an underestimation of the effect size of the relationship between racism and health, as people in the comparison group (i.e. did not experience racism in the last 12 months) may have experienced racism more than 12 months ago. Finally, our study focuses on racial discrimination and does not consider other forms of discrimination (e.g. on the basis of age, gender, class etc.) that may operate concurrently and cumulatively to negatively affect health and wellbeing [62].

While the racism questions are consistent within repeats of each of the two surveys (i.e. NZHS and GSS), the questions are not identical in these two data sources. The major distinction is that they ask about experience of racism in two commonly used but distinct approaches [4, 48, 69]: a one-step question (the NZHS, where respondents are asked about racial discrimination in each of five domains) and a two-step question (the GSS, where respondents are asked about any discrimination over the last year, and if answering yes are asked about the type of discrimination and where it happened). Research has shown that where questions are directly comparable with regards to the form or setting of discrimination, the two-step question tends to produce lower estimates of racism than the one-step question [4, 48, 69]. However, the two surveys used in our study (NZHS and GSS) have limited comparability at the individual discrimination setting or item level because of differences in question wording and in the number of settings or forms of racial discrimination asked about. However, at the broader level of overall racial discrimination, outputs for each survey type appear to be consistent for analyses of the surveys examined in this study. In addition, both question types show associations between reported experience of racism and health measures in the wider literature [1, 4, 69].

Our study focuses on experience of racism measured at the interpersonal level. As such it does not capture racism at the institutional or structural level well. Associations between individual experience of racism and our outcomes were adjusted for a number of covariates, including socioeconomic measures. Socioeconomic position (SEP) is highly patterned by ethnicity in New Zealand with large inequities between European and non-European ethnic groups [13, 70, 71]. It is important to note that this ethnic patterning of SEP can be conceptualised as a marker of institutionalised racism and that experience of racism at the individual level and institutional levels are inter-related [1, 16, 72, 73]. Adjustment for SEP in the analyses demonstrates the independent effect of individual experience of racism on health/wellbeing. Examining how SEP operates as both a mediator and a risk factor for increased exposure to experience of racism warrants further study in longitudinal research, which is better able to tease out the time sequence of events and hence allow conduct of more formally robust mediation analyses [74].

While the use of meta-analysis techniques allowed for improved precision of effect sizes for the impact of racism when stratified by ethnicity, compared to considering a single survey analysis in isolation, these stratified estimates were still limited by smaller numbers and hence poorer precision of estimates. This also limited our ability to undertake more in-depth analysis of the patterning of racism within ethnic groups by other variables. This has implications for survey design, where it is important to sample populations with higher needs adequately. In New Zealand, this means adequately sampling smaller ethnic groups in order to provide robust evidence by ethnicity.

This study provides a comprehensive overview of the experience of racism using nationally collected survey data from the NZHS and GSS over a ten-year period. It extends previous analyses of these studies to focus on recent experience of racism and its link to several distinct negative health and wellbeing measures. It also utilises meta-analysis techniques to improve precision for estimates, which is of particular use for analytical questions that were previously limited by small respondent numbers. Racism is a broad system and requires multiple methods to measure and intervene to not only improve health and reduce ethnic health inequities but also to improve social wellbeing and reduce inequities more broadly. While further research and different study designs are necessary to understand specific mechanisms, causal health effects, and the effectiveness of specific interventions, the on-going monitoring of racism as a determinant of health at a population level remains a key component of understanding, prioritising and addressing racism as not only a health, but also a human rights issue.

## Supporting information

S1 Fig Prevalence of experience of racism (last 12 months) by setting and ethnicity, GSS 2008, 2010, 2012. Figure note: Data is from the Statistics New Zealand data lab.
(TIFF)

S2 Fig Association between experience of racial discrimination and health and wellbeing measures by survey and combined in meta-analysis (unadjusted). (DOCX)

S3 Fig Association between experience of racial discrimination (last 12 months) and poor/fair self-rated by survey, stratified by ethnicity and adjusted for age, gender, nativity, NZDep, education qualification. (TIFF)

S4 Fig Association between experience of racial discrimination (last 12 months) and life satisfaction (dissatisfied/very dissatisfied life satisfaction) by survey, stratified by ethnicity and adjusted for age, gender, nativity, NZDep, education qualification. (TIFF)

S5 Fig Association between experience of racial discrimination (last 12 months) and SF12 mental health summary score by survey, stratified by ethnicity and adjusted for age, gender, nativity, NZDep, education qualification. (TIFF)

S6 Fig Association between experience of racial discrimination (last 12 months) and SF12 physical health summary score by survey, stratified by ethnicity and adjusted for age, gender, nativity, NZDep, education qualification. (TIFF)

S1 Table Survey questions used to examine racial discrimination. (DOCX)

S2 Table Prevalence of reported experience of any racism in the last 12 months by ethnicity according to survey type and survey year (data for Fig 1). Table notes: **A**. Unweighted frequency gives total number of respondents answering yes to recent experience of racism. **B**. Unweighted frequency gives total number of respondents for total Māori, Pacific, Asian ethnic groups and residual European/Other group. **C**. Weighted prevalence gives estimated prevalence of recent experience of racism for NZ adult population (95% CI further accounts for stratification and clustering of responses).
(DOCX)

S3 Table Patterning of experience of racial discrimination (last 12 months) by sociodemographic factors. Results of multivariable logistic regression analysis by survey year. Table note: Ethnicity is prioritised in the following order: Māori, Pacific, Asian, European/Other. ^a^Māori overseas born are included in the model but data is not shown because of small numbers.
(DOCX)

S4 Table Prevalence of experience of racism (last 12 months) by setting and ethnicity, GSS 2008, 2010, 2012. Table note: Percentages are weighted to give population prevalences. Total ethnicity is used for Māori, Pacific and Asian groups, with a mutually exclusive European/Other comparator group. Data is from the Statistics New Zealand data lab and conforms to Statistics New Zealand data lab rules with rounding to 3 of raw freqencies and suppression of cells with small numbers (Statistics New Zealand (2015). Microdata output guide (Third edition). Wellington, Statistics New Zealand).
(DOCX)

S5 Table Unadjusted association between experience of racial discrimination (last 12 months) and health and wellbeing measures, ethnically stratified models by survey and random effects outputs from meta-analysis (NZHS 2002/03, 2006/07, 20011/12, GSS 2008, 2010, 2012). Notes: OR = Odds ratio; MD = mean difference; ^a^data only available for GSSs; ^b^NZHS 2002/03 not analysed because used an earlier version of SF-12; prioritised ethnicity.
(DOCX)
