# Supplementary figures and images for: Racism and health in New Zealand: Prevalence over time and associations between recent experience of racism and health and wellbeing measures using national survey data
Source: PLoS One. 2018 May 3;13(5):e0196476. doi: 10.1371/journal.pone.0196476 (PMC5933753; doi:10.1371/journal.pone.0196476)

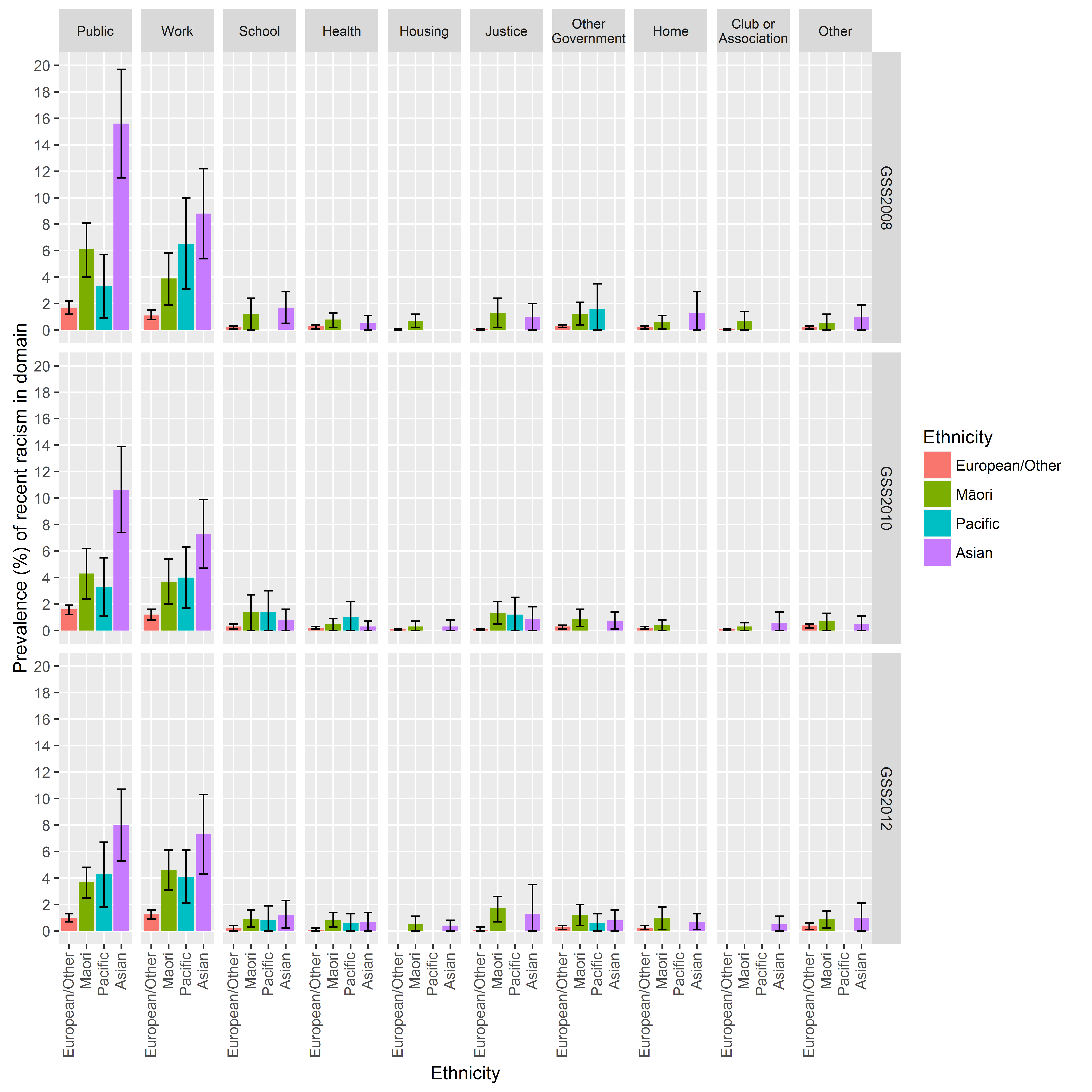

Supplement: S1 Fig — Figure note: Data is from the Statistics New Zealand data lab. (TIFF) [file pone.0196476.s001.tiff]
