# Supplementary material for: Racism and health in New Zealand: Prevalence over time and associations between recent experience of racism and health and wellbeing measures using national survey data
Source: PLoS One. 2018 May 3;13(5):e0196476. doi: 10.1371/journal.pone.0196476 (PMC5933753; doi:10.1371/journal.pone.0196476)
Supplement: S2 Fig — (DOCX) [file pone.0196476.s002.docx]

**S2 Figure: Association between experience of racial discrimination and health and wellbeing measures by survey and combined in meta-analysis (unadjusted)**

Impact of racism on self-rated health (odds of poor/fair health with exposure)
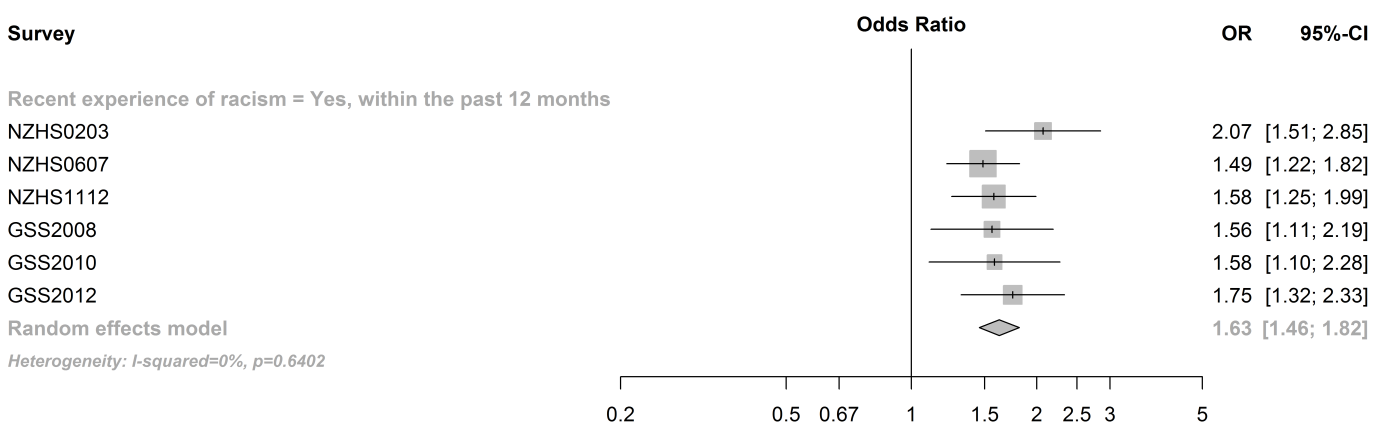


Impact of racism on wellbeing (odds of reporting dissatisfied/very dissatisfied with exposure)
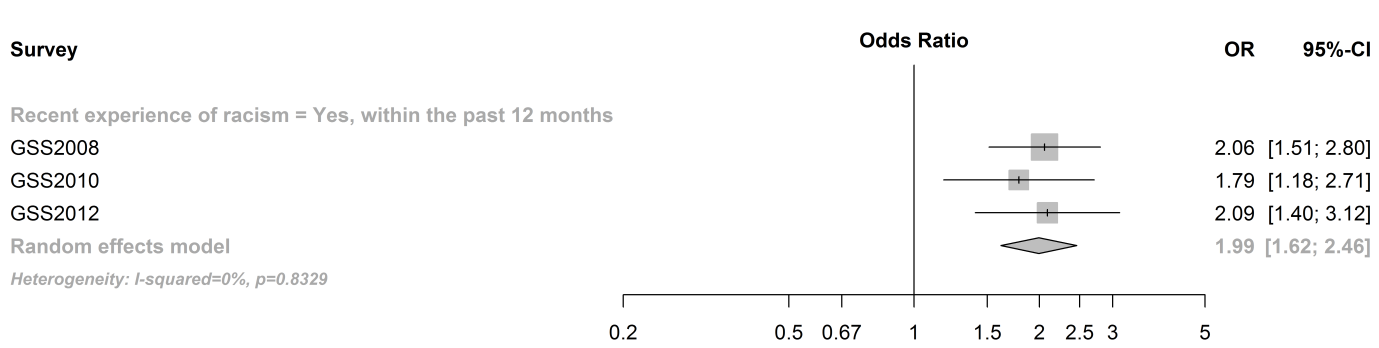


Impact of racism on SF12 mental health (negative scores indicate worse health with exposure)
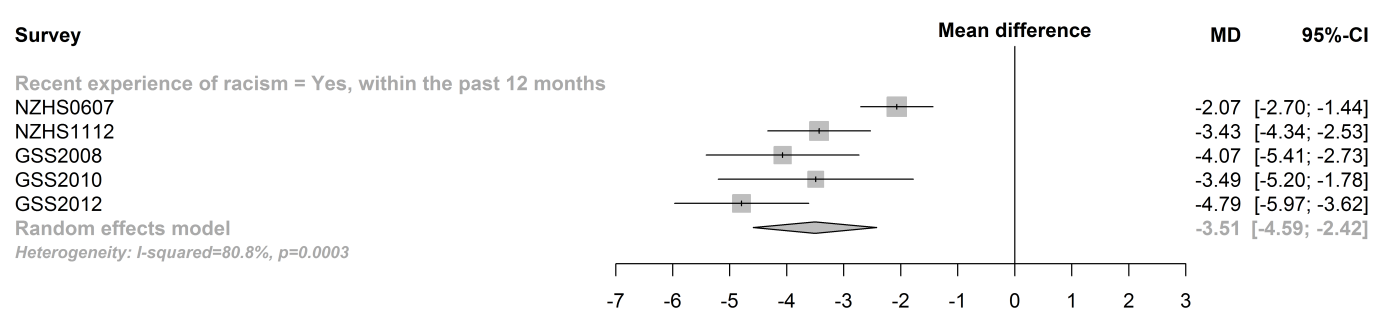


Impact of racism on SF12 physical health (negative scores indicate worse health with exposure)**
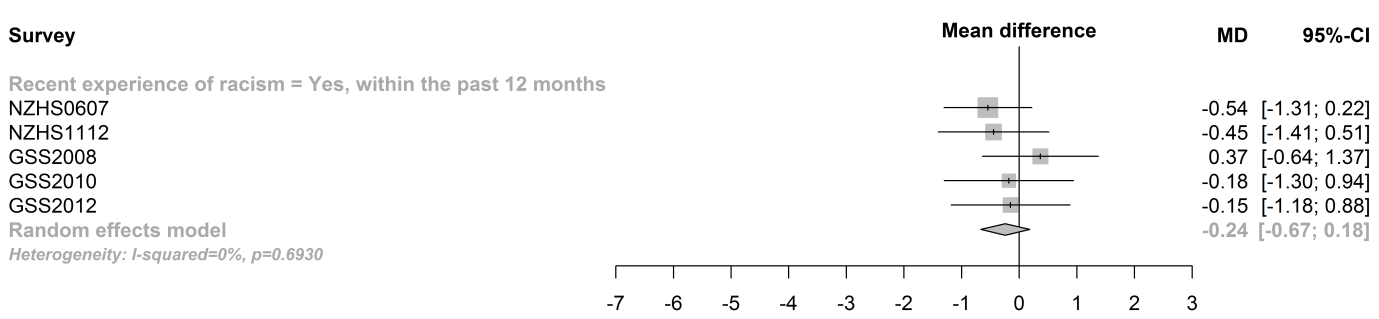
**
