# Supplementary material for: Racism and health in New Zealand: Prevalence over time and associations between recent experience of racism and health and wellbeing measures using national survey data
Source: PLoS One. 2018 May 3;13(5):e0196476. doi: 10.1371/journal.pone.0196476 (PMC5933753; doi:10.1371/journal.pone.0196476)
Supplement: S1 Table — (DOCX) [file pone.0196476.s007.docx]

**S1 Table: Survey questions used to examine racial discrimination**

**S1a Table: Self-reported experience of racial discrimination questions 2002/03, 2006/07, 2011/12 New Zealand Health Surveys**

| **Item** | **Survey** | **Question number** | **Question wording** | **Response options** |
| --- | --- | --- | --- | --- |
| Physical  and/or  Verbal attack | 2002/03 | Q. 270 | Have you ever been a victim of an **ethnically** motivated attack (verbal or physical abuse to the person or property) in New Zealand? ***(Multiple possible)*** *(Card 270)* | Yes, verbal – within the past 12 months  Yes, verbal – more than 12 months ago  Yes, physical – within the past 12 months  Yes, physical – more than 12 months ago  No  Don’t know/unsure  Refuse/d  Unsure not used in 2011/12, just Don’t know |
|  | 2006/07 | 5.09 | [Showcard 5.09]  Have you ever been a victim of an ethnically motivated attack (verbal or physical abuse to the person or property) *in New Zealand*? Please look at card 5.09. [Circle all mentioned] |  |
|  | 2011/12 | 5.10 | [Showcard]  Have you ever been a victim of an ethnically motivated attack (verbal or physical abuse to the person or property) in New Zealand? [Multiple responses possible] |  |
| Unfair treatment (health) | 2002/03 | Q. 271 | Have you ever been treated unfairly (e.g. treated differently, kept waiting) by a health professional (e.g., doctor, nurse, dentist etc) **because of your ethnicity** in New Zealand? *(Card 271)* | [single response only]  Yes, within the past 12 months  Yes, more than 12 months ago  No  Don’t know/unsure  Refuse/d  Unsure not used in 2011/12, just Don’t know |
|  | 2006/07 | 5.10 | [Showcard 5.10]  Have you ever been treated unfairly (for example, kept waiting or treated differently) by a health professional (that is, a doctor, nurse, dentist etc) because of your ethnicity in New Zealand?  [Circle one] |  |
|  | 2011/12 | 5.11 | [Showcard]  Have you ever been treated unfairly (for example, kept waiting or treated differently) by a health professional (that is, a doctor, nurse, dentist etc) because of your ethnicity in New Zealand? |  |
| Unfair treatment (work) | 2002/03 | Q. 272 | Have you ever been treated unfairly at work or been refused a job **because of your ethnicity** in New Zealand? *(Card 271)* |  |
|  | 2006/07 | 5.11 | [Showcard 5.10]  Have you ever been treated unfairly at work or been refused a job because of your ethnicity in New Zealand?  [Circle one] |  |
|  | 2011/12 | 5.12 | [Showcard]  Have you ever been treated unfairly at work or been refused a job because of your ethnicity in New Zealand? |  |
| Unfair treatment (housing) | 2002/03 | Q. 273 | Have your ever been treated unfairly when renting or buying housing **because of your ethnicity** in New Zealand? *(Card 271)* |  |
|  | 2006/07 | 5.12 | [Showcard 5.10]  Have your ever been treated unfairly when renting or buying housing because of your ethnicity in New Zealand?  [Circle one] |  |
|  | 2011/12 | 5.13 | [Showcard]  Have you ever been treated unfairly when renting or buying housing because of your ethnicity in New Zealand? |  |

Notes: interviewer instructions are in blue, minor changes to surveys are in red.

Sources: Questionnaires are available at [www.moh.govt.nz](http://www.moh.govt.nz)

**S1b Table: Self-reported experience of discrimination questions NZ General Social Surveys 2008, 2012, 2012**

| **Item** | **Survey** | **Question number** | **Question wording** | **Response options** |
| --- | --- | --- | --- | --- |
| Discrimination | 2008, 2010, 2012 | HUMQ05 | The next question is about discrimination in New Zealand.  In the last 12 months, have you been treated unfairly or had something nasty done to you because of the group you belong to or seem to belong to? | 01 yes (go to HUMQ06)  02 no (skip to next section)  88 don’t know (skip to next section)  99 refused (skip to next section) |
| Setting of discrimination | 2008, 2010, 2012 | HUMQ07 | *Showcard 57*  *Select all that apply*  Looking at showcard 57, what situation/s you were in when you were discriminated against? You can choose as many as you need.  *(For each answer, go to HUMS1Q01 to select basis of discrimination)* | 11. at home  12. at work or while working  13. on the street or in a public place of any kind  14. using transport of any kind  15. getting service when buying something  16. getting into a school or other place of learning, or being treated fairly there  17. joining an association or club of any kind  18. applying for or keeping a job or position  19. applying for or keeping a flat or housing of any kind  20. dealing with the police  21. dealing with the courts  22. dealing with other government officials  23. dealing with people involved in health care  24. other – please specify  88. don’t know  99. refused |
| Basis of discrimination | 2008, 2010, 2012 | HUMS1Q01 | *Showcard 58*  *Select all that apply*  Looking at showcard 58, why do you think people discriminated against you when you were in that situation/those situations? You can choose as many as you need. | 11. my skin colour  12. my nationality, race or ethnic group  13. the language I speak  14. the way I dress or my appearance  15. my gender (male or female)  16. my age  17. a disability or health issue I have  18. my marital status (whether or not you are married or living with someone)  19. my family status (whether or not you have children)  20. my sexual orientation (lesbian/gay/straight/bisexual/transgender)  21. what I do for a job  22. my religious beliefs  23. my political position  24. other – please specify (go to HUMS1Q01Oth)  88. don’t know  99. refused |

Note: italicised font are instructions to interviewers

Sources: Questionnaires are available at <https://cdm20045.contentdm.oclc.org/digital/collection/p20045coll2/search/searchterm/GSS>
