# Supplementary material for: Racism and health in New Zealand: Prevalence over time and associations between recent experience of racism and health and wellbeing measures using national survey data
Source: PLoS One. 2018 May 3;13(5):e0196476. doi: 10.1371/journal.pone.0196476 (PMC5933753; doi:10.1371/journal.pone.0196476)
Supplement: S2 Table — Table notes: A. Unweighted frequency gives total number of respondents answering yes to recent experience of racism. B. Unweighted frequency gives total number of respondents for total Māori, Pacific, Asian ethnic groups and residual European/Other group. C. Weighted prevalence gives estimated prevalence of recent experience of racism for NZ adult population (95% CI further accounts for stratification and clustering of responses). (DOCX) [file pone.0196476.s008.docx]

**S2 Table: Prevalence of reported experience of any racism in the last 12 months by ethnicity according to survey type and survey year (data for figure 1)**

| **Survey** | **Ethnicity** | **Year** | **Unweighted^a^ N reporting experience of racsim** | **Unweighted^b^ total N in group** | **Weighted^c^ prevalence (95% CI)** |
| --- | --- | --- | --- | --- | --- |
|  |  |  |  |  |  |
| NZHS |  |  |  |  |  |
|  | Māori | 02/03 | 488 | 3945 | 14.2 (11.9, 16.6) |
|  |  | 06/07 | 385 | 3154 | 12.2 (10.7, 13.7) |
|  |  | 11/12 | 234 | 2547 | 9.3 (7.8, 10.9) |
|  |  |  |  |  |  |
|  | Pacific | 02/03 | 90 | 940 | 9.5 (6.8, 12.3) |
|  |  | 06/07 | 117 | 1031 | 11.1 (8.8, 13.4) |
|  |  | 11/12 | 76 | 927 | 9.2 (6.7, 11.7) |
|  |  |  |  |  |  |
|  | Asian | 02/03 | 171 | 1013 | 20.2 (16.4, 23.9) |
|  |  | 06/07 | 271 | 1509 | 18.6 (16, 21.1) |
|  |  | 11/12 | 119 | 900 | 13.2 (10.7, 15.6) |
|  |  |  |  |  |  |
|  | European/Other | 02/03 | 299 | 6220 | 4.9 (4.2, 5.5) |
|  |  | 06/07 | 305 | 6940 | 4.7 (4.1, 5.4) |
|  |  | 11/12 | 279 | 8229 | 3.3 (2.8, 3.8) |
|  |  |  |  |  |  |
| GSS |  |  |  |  |  |
|  | Māori | 2008 | 102 | 968 | 10.3 (7, 13.5) |
|  |  | 2010 | 92 | 947 | 9.4 (6.7, 12.2) |
|  |  | 2012 | 107 | 1113 | 9.8 (7.7, 11.9) |
|  |  |  |  |  |  |
|  | Pacific | 2008 | 37 | 378 | 8.8 (5, 12.7) |
|  |  | 2010 | 30 | 289 | 8.3 (4.6, 12) |
|  |  | 2012 | 42 | 420 | 8.1 (5.3, 11) |
|  |  |  |  |  |  |
|  | Asian | 2008 | 109 | 535 | 21.3 (16.7, 26) |
|  |  | 2010 | 94 | 566 | 15.3 (11.9, 18.7) |
|  |  | 2012 | 96 | 646 | 13.5 (9.9, 17) |
|  |  |  |  |  |  |
|  | European/Other | 2008 | 188 | 6853 | 2.9 (2.3, 3.5) |
|  |  | 2010 | 216 | 6772 | 3.3 (2.8, 3.8) |
|  |  | 2012 | 184 | 6326 | 2.9 (2.3, 3.4) |
|  |  |  |  |  |  |

a Unweighted frequency gives total number of respondents answering yes to recent experience of racism

b, Unweighted frequency gives total number of respondents for total Māori, Pacific, Asian ethnic groups and residual European/Other group

c Weighted prevalence gives estimated prevalence of recent experience of racism for NZ adult population (95% CI further accounts for stratification and clustering of responses)
