# Supplementary material for: Racism and health in New Zealand: Prevalence over time and associations between recent experience of racism and health and wellbeing measures using national survey data
Source: PLoS One. 2018 May 3;13(5):e0196476. doi: 10.1371/journal.pone.0196476 (PMC5933753; doi:10.1371/journal.pone.0196476)
Supplement: S3 Table — Table note: Ethnicity is prioritised in the following order: Māori, Pacific, Asian, European/Other. aMāori overseas born are included in the model but data is not shown because of small numbers. (DOCX) [file pone.0196476.s009.docx]

**S3 Table: Patterning of experience of racial discrimination (last 12 months) by sociodemographic factors. Results of multivariable logistic regression analysis by survey year.**

| Variable | Level | |  |  |  | Recent experience of racism | |  |  |
| --- | --- | --- | --- | --- | --- | --- | --- | --- | --- |
|  |  |  |  |  |  | Odds ratio (95% CI) | |  |  |
|  |  |  |  | GSS 2008 | GSS 2010 | GSS 2012 | NZHS 02/03 | NZHS 06/07 | NZHS 11/12 |
|  |  |  |  |  |  |  |  |  |  |
| Ethnicity/ | European/Other | NZ born |  | 1 (ref) | 1 (ref) | 1 (ref) | 1 (ref) | 1 (ref) | 1 (ref) |
| Nativity |  | Overseas born |  | 2.09 (1.45, 3.01) | 1.87 (1.22, 2.86) | 3.50 (2.35, 5.21) | 1.95 (1.40, 2.72) | 1.50 (1.10, 2.05) | 2.68 (1.88, 3.80) |
|  | Māori | NZ born |  | 4.08 (2.44, 6.83) | 3.51 (2.28, 5.40) | 4.50 (3.29, 6.15) | 2.60 (1.93, 3.49) | 2.29 (1.80, 2.91) | 2.68 (2.02, 3.57) |
|  |  | Overseas born* |  | *Not reported^a^* | *Not reported^a^* | *Not reported^a^* | *Not reported^a^* | *Not reported^a^* | *Not reported^a^* |
|  | Pacific | NZ born |  | 2.33 (0.93, 5.83) | 3.09 (1.33, 7.21) | 3.29 (1.51, 7.18) | 1.04 (0.52, 2.07) | 1.87 (1.21, 2.87) | 1.76 (0.98, 3.14) |
|  |  | Overseas born |  | 4.34 (2.17, 8.71) | 2.44 (1.08, 5.51) | 2.29 (1.24, 4.25) | 1.32 (0.80, 2.18) | 1.76 (1.19, 2.59) | 2.86 (1.80, 4.55) |
|  | Asian | NZ born |  | 5.30 (1.45, 19.33) | 1.47 (0.41, 5.27) | 7.83 (2.19, 27.98) | 1.19 (0.35, 4.02) | 2.46 (1.07, 5.63) | 2.19 (0.68, 7.06) |
|  |  | Overseas born |  | 9.39 (5.95, 14.84) | 5.81 (4.10, 8.22) | 6.59 (4.56, 9.53) | 4.77 (3.48, 6.54) | 4.20 (3.30, 5.35) | 5.16 (3.87, 6.87) |
|  |  |  |  |  |  |  |  |  |  |
| Age group |  | 15–24 |  | 1 (ref) | 1 (ref) | 1 (ref) | 1 (ref) | 1 (ref) | 1 (ref) |
|  |  | 25–34 |  | 0.72 (0.50, 1.02) | 0.99 (0.65, 1.50) | 1.08 (0.68, 1.73) | 0.58 (0.43, 0.78) | 0.90 (0.69, 1.19) | 0.84 (0.59, 1.20) |
|  |  | 35–44 |  | 0.68 (0.48, 0.96) | 1.18 (0.83, 1.67) | 1.21 (0.74, 1.98) | 0.62 (0.46, 0.84) | 0.84 (0.64, 1.10) | 0.97 (0.68, 1.37) |
|  |  | 45–54 |  | 0.75 (0.47, 1.19) | 1.12 (0.69, 1.83) | 1.52 (0.97, 2.40) | 0.47 (0.32, 0.68) | 0.65 (0.48, 0.88) | 0.91 (0.63, 1.31) |
|  |  | 55–64 |  | 0.56 (0.34, 0.93) | 0.68 (0.47, 1.01) | 0.77 (0.47, 1.25) | 0.34 (0.22, 0.51) | 0.54 (0.38, 0.75) | 0.86 (0.58, 1.29) |
|  |  | 65–74 |  | 0.28 (0.14, 0.53) | 0.29 (0.16, 0.54) | 0.73 (0.41, 1.33) | 0.16 (0.08, 0.31) | 0.19 (0.11, 0.31) | 0.30 (0.17, 0.52) |
|  |  | 75+ |  | 0.08 (0.02, 0.29) | 0.14 (0.05, 0.43) | 0.08 (0.03, 0.27) | 0.14 (0.07, 0.28) | 0.19 (0.10, 0.36) | 0.10 (0.04, 0.23) |
|  |  |  |  |  |  |  |  |  |  |
| Gender |  | Male |  | 1.25 (0.96, 1.63) | 1.09 (0.82, 1.44) | 1.19 (0.91, 1.57) | 1.22 (0.99, 1.51) | 1.25 (1.05, 1.49) | 1.10 (0.90, 1.35) |
|  |  | Female |  | 1 (ref) | 1 (ref) | 1 (ref) | 1 (ref) | 1 (ref) | 1 (ref) |
|  |  |  |  |  |  |  |  |  |  |
| Education | No secondary qualification | |  | 0.77 (0.55, 1.09) | 0.80 (0.53, 1.20) | 1.04 (0.71, 1.50) | 1.10 (0.84, 1.43) | 1.03 (0.83, 1.27) | 1.14 (0.89, 1.46) |
|  | Secondary qualification | |  | 1 (ref) | 1 (ref) | 1 (ref) | 1 (ref) | 1 (ref) | 1 (ref) |
|  |  |  |  |  |  |  |  |  |  |

**S3 Table (continued): Patterning of experience of racial discrimination (last 12 months) by sociodemographic factors. Results of multivariable logistic regression analysis by survey year**

| Variable | Level | |  |  |  | Recent experience of racism | |  |  |
| --- | --- | --- | --- | --- | --- | --- | --- | --- | --- |
|  |  |  |  |  |  | Odds ratio (95% CI) | |  |  |
|  |  |  |  | GSS 2008 | GSS 2010 | GSS 2012 | NZHS 02/03 | NZHS 06/07 | NZHS 11/12 |
|  |  |  |  |  |  |  |  |  |  |
| NZDep |  | 1 |  | 1 (ref) | 1 (ref) | 1 (ref) | 1 (ref) | 1 (ref) | 1 (ref) |
| Quintile |  | 2 |  | 1.28 (0.82, 2.02) | 1.06 (0.66, 1.72) | 1.09 (0.65, 1.80) | 1.75 (1.13, 2.70) | 1.32 (0.90, 1.93) | 0.80 (0.52, 1.23) |
|  |  | 3 |  | 1.09 (0.70, 1.69) | 1.29 (0.85, 1.95) | 1.42 (0.91, 2.21) | 1.53 (0.99, 2.38) | 1.82 (1.27, 2.60) | 1.39 (0.94, 2.07) |
|  |  | 4 |  | 1.87 (1.18, 2.97) | 1.04 (0.64, 1.68) | 1.83 (1.18, 2.82) | 1.74 (1.16, 2.63) | 1.75 (1.23, 2.48) | 1.21 (0.83, 1.78) |
|  |  | 5 |  | 1.03 (0.60, 1.76) | 0.96 (0.59, 1.58) | 1.73 (1.14, 2.63) | 2.21 (1.45, 3.36) | 2.00 (1.39, 2.89) | 2.07 (1.43, 3.01) |
|  |  |  |  |  |  |  |  |  |  |

Note: Ethnicity is prioritised in the following order: Māori, Pacific, Asian, European/Other. ^a^Māori overseas born are included in the model but data is not shown because of small numbers.
