# Supplementary material for: Racism and health in New Zealand: Prevalence over time and associations between recent experience of racism and health and wellbeing measures using national survey data
Source: PLoS One. 2018 May 3;13(5):e0196476. doi: 10.1371/journal.pone.0196476 (PMC5933753; doi:10.1371/journal.pone.0196476)
Supplement: S4 Table — Table note: Percentages are weighted to give population prevalences. Total ethnicity is used for Māori, Pacific and Asian groups, with a mutually exclusive European/Other comparator group. Data is from the Statistics New Zealand data lab and conforms to Statistics New Zealand data lab rules with rounding to 3 of raw freqencies and suppression of cells with small numbers (Statistics New Zealand (2015). Microdata output guide (Third edition). Wellington, Statistics New Zealand). (DOCX) [file pone.0196476.s010.docx]

**S4 Table: Prevalence of experience of racism (last 12 months) by setting and ethnicity, GSS 2008, 2010, 2012**

|  | **European/other** | | **Asian** | | **Māori** | | **Pacific** | |
| --- | --- | --- | --- | --- | --- | --- | --- | --- |
|  | **%** | **(95% CI)** | **%** | **(95% CI)** | **%** | **(95% CI)** | **%** | **(95% CI)** |
|  |  |  |  |  |  |  |  |  |
| **2008** |  |  |  |  |  |  |  |  |
| Club/association | 0.1 | (0.0, 0.1) | - | - | 0.7 | (0.0, 1.4) | - | - |
| Other government | 0.3 | (0.2, 0.4) | - | - | 1.2 | (0.4, 2.1) | 1.6 | (0.0, 3.5) |
| Health | 0.3 | (0.1, 0.4) | 0.5 | (0.0, 1.1) | 0.8 | (0.2, 1.3) | - | - |
| Home | 0.2 | (0.1, 0.3) | 1.3 | (0.0, 2.9) | 0.6 | (0.1, 1.1) | - | - |
| Housing | 0.0 | (0.0, 0.1) | - | - | 0.7 | (0.2, 1.2) | - | - |
| Justice | 0.1 | (0.0, 0.1) | 1.0 | (0.0, 2.0) | 1.3 | (0.2, 2.4) | - | - |
| Public | 1.7 | (1.2, 2.2) | 15.6 | (11.5, 19.7) | 6.1 | (4.0, 8.1) | 3.3 | (0.9, 5.7) |
| School | 0.2 | (0.1, 0.3) | 1.7 | (0.5, 2.9) | 1.2 | (0.0, 2.4) | - | - |
| Work | 1.1 | (0.8, 1.5) | 8.8 | (5.4, 12.2) | 3.9 | (1.9, 5.8) | 6.5 | (3.1, 10.0) |
| Other | 0.2 | (0.1, 0.3) | 1.0 | (0.0, 1.9) | 0.5 | (0.0, 1.2) | - | - |
|  |  |  |  |  |  |  |  |  |
| **2010** |  |  |  |  |  |  |  |  |
| Club/association | 0.1 | (0.0, 0.1) | 0.6 | (0.0, 1.4) | 0.3 | (0.0, 0.6) | - | - |
| Other government | 0.3 | (0.1, 0.4) | 0.7 | (0.1, 1.4) | 0.9 | (0.3, 1.6) | - | - |
| Health | 0.2 | (0.1, 0.3) | 0.3 | (0.0, 0.7) | 0.5 | (0.0, 0.9) | 1.0 | (0.0, 2.2) |
| Home | 0.2 | (0.1, 0.3) | - | - | 0.4 | (0.0, 0.8) | - | - |
| Housing | 0.1 | (0.0, 0.1) | 0.3 | (0.0, 0.8) | 0.3 | (0.0, 0.7) | - | - |
| Justice | 0.1 | (0.0, 0.1) | 0.9 | (0.0, 1.8) | 1.3 | (0.5, 2.2) | 1.2 | (0.0, 2.5) |
| Public | 1.6 | (1.2, 1.9) | 10.6 | (7.4, 13.9) | 4.3 | (2.4, 6.2) | 3.3 | (1.1, 5.5) |
| School | 0.3 | (0.1, 0.5) | 0.8 | (0.0, 1.6) | 1.4 | (0.0, 2.7) | 1.4 | (0.0, 3.0) |
| Work | 1.2 | (0.8, 1.6) | 7.3 | (4.7, 9.9) | 3.7 | (2.0, 5.4) | 4.0 | (1.7, 6.3) |
| Other | 0.4 | (0.2, 0.5) | 0.5 | (0.0, 1.1) | 0.7 | (0.0, 1.3) | - | - |
|  |  |  |  |  |  |  |  |  |
| **2012** |  |  |  |  |  |  |  |  |
| Club/association | - | - | 0.5 | (0.0, 1.1) | - | - | - | - |
| Other government | 0.3 | (0.1, 0.4) | 0.8 | (0.0, 1.6) | 1.2 | (0.4, 2.0) | 0.6 | (0.0, 1.3) |
| Health | 0.1 | (0.0, 0.2) | 0.7 | (0.0, 1.4) | 0.8 | (0.3, 1.4) | 0.6 | (0.0, 1.3) |
| Home | 0.2 | (0.1, 0.4) | 0.7 | (0.1, 1.3) | 1.0 | (0.1, 1.8) | - | - |
| Housing | - | - | 0.4 | (0.0, 0.8) | 0.5 | (0.0, 1.1) | - | - |
| Justice | 0.1 | (0.0, 0.3) | 1.3 | (0.0, 3.5) | 1.7 | (0.7, 2.6) | - | - |
| Public | 1.0 | (0.7, 1.3) | 8.0 | (5.3, 10.7) | 3.7 | (2.5, 4.8) | 4.3 | (1.8, 6.7) |
| School | 0.2 | (0.0, 0.4) | 1.2 | (0.2, 2.3) | 0.9 | (0.3, 1.6) | 0.8 | (0.0, 1.9) |
| Work | 1.3 | (0.9, 1.6) | 7.3 | (4.3, 10.3) | 4.6 | (3.1, 6.1) | 4.1 | (2.1, 6.1) |
| Other | 0.4 | (0.1, 0.6) | 1.0 | (0.0, 2.1) | 0.9 | (0.2, 1.5) | - | - |
|  |  |  |  |  |  |  |  |  |

Note: Percentages are weighted to give population prevalences. Total ethnicity is used for Māori, Pacific and Asian groups, with a mutually exclusive European/Other comparator group. Data is from the Statistics New Zealand data lab and conforms to Statistics New Zealand data lab rules with rounding to 3 of raw freqencies and suppression of cells with small numbers (Statistics New Zealand (2015). Microdata output guide (Third edition). Wellington, Statistics New Zealand).
