# Supplementary material for: Racism and health in New Zealand: Prevalence over time and associations between recent experience of racism and health and wellbeing measures using national survey data
Source: PLoS One. 2018 May 3;13(5):e0196476. doi: 10.1371/journal.pone.0196476 (PMC5933753; doi:10.1371/journal.pone.0196476)
Supplement: S5 Table — Notes: OR = Odds ratio; MD = mean difference; adata only available for GSSs; bNZHS 2002/03 not analysed because used an earlier version of SF-12; prioritised ethnicity. (DOCX) [file pone.0196476.s011.docx]

| **Ethnicity** | **Survey** |  | **Self-rated health** | |  | **Life satisfaction^a^** | |  | **SF12 mental health score^b^** | |  | **SF12 physical health score^b^** | |
| --- | --- | --- | --- | --- | --- | --- | --- | --- | --- | --- | --- | --- | --- |
|  |  |  | **OR** | **(95% CI)** |  | **OR** | **(95% CI)** |  | **MD** | **(95% CI)** |  | **MD** | **(95% CI)** |
|  |  |  |  |  |  |  |  |  |  |  |  |  |  |
| **Māori** | NZHS 2002/03 |  | 1.55 | (0.85, 2.84) |  |  |  |  |  |  |  |  |  |
|  | NZHS 2006/07 |  | 1.80 | (1.28, 2.53) |  |  |  |  | -3.30 | (-4.57, -2.03) |  | -1.32 | (-2.90, 0.26) |
|  | NZHS 2011/12 |  | 1.72 | (1.11, 2.68) |  |  |  |  | -5.10 | (-7.08, -3.12) |  | -0.55 | (-2.08, 0.99) |
|  | GSS 2008 |  | 1.65 | (0.99, 2.74) |  | 1.70 | (0.79, 3.67) |  | -5.16 | (-8.59, -1.72) |  | 0.02 | (-2.17, 2.20) |
|  | GSS 2010 |  | 1.11 | (0.40, 3.09) |  | 0.91 | (0.45, 1.84) |  | -3.72 | (-6.74, -0.70) |  | 1.73 | (-0.38, 3.85) |
|  | GSS 2012 |  | 2.51 | (1.44, 4.39) |  | 1.48 | (0.65, 3.41) |  | -5.44 | (-7.83, -3.05) |  | -1.93 | (-3.87, 0.01) |
|  | **Pooled estimate** |  | **1.77** | **(1.45, 2.17)** |  | **1.28** | **(0.82, 1.99)** |  | **-4.16** | **(-5.08, -3.23)** |  | **-0.50** | **(-1.64, 0.63)** |
|  |  |  |  |  |  |  |  |  |  |  |  |  |  |
| **Pacific** | NZHS 2002/03 |  | 0.69 | (0.21, 2.25) |  |  |  |  |  |  |  |  |  |
|  | NZHS 2006/07 |  | 1.86 | (1.01, 3.41) |  |  |  |  | -2.61 | (-4.78, -0.45) |  | -2.17 | (-4.52, 0.18) |
|  | NZHS 2011/12 |  | 2.10 | (1.03, 4.30) |  |  |  |  | -6.53 | (-10.27, -2.79) |  | -1.73 | (-4.77, 1.31) |
|  | GSS 2008 |  | 0.59 | (0.08, 4.45) |  | 2.69 | (0.48, 15.19) |  | -2.27 | (-7.10, 2.56) |  | 0.06 | (-5.17, 5.29) |
|  | GSS 2010 |  | 1.10 | (0.13, 9.45) |  | 2.19 | (0.24, 20.25) |  | -5.46 | (-11.20, 0.29) |  | 0.42 | (-3.44, 4.28) |
|  | GSS 2012 |  | 1.07 | (0.34, 3.32) |  | 1.15 | (0.28, 4.75) |  | -3.55 | (-8.14, 1.04) |  | -2.06 | (-6.48, 2.35) |
|  | **Pooled estimate** |  | **1.53** | **(1.04, 2.25)** |  | **1.72** | **(0.64, 4.59)** |  | **-3.59** | **(-5.16, -2.02)** |  | **-1.48** | **(-2.98, 0.02)** |
|  |  |  |  |  |  |  |  |  |  |  |  |  |  |
| **Asian** | NZHS 2002/03 |  | 1.43 | (0.53, 3.88) |  |  |  |  |  |  |  |  |  |
|  | NZHS 2006/07 |  | 1.27 | (0.83, 1.93) |  |  |  |  | -1.82 | (-3.12, -0.53) |  | 0.56 | (-0.70, 1.81) |
|  | NZHS 2011/12 |  | 0.64 | (0.31, 1.34) |  |  |  |  | -2.26 | (-3.71, -0.80) |  | 0.33 | (-1.27, 1.93) |
|  | GSS 2008 |  | 0.93 | (0.27, 3.22) |  | 1.60 | (0.69, 3.69) |  | -3.49 | (-6.26, -0.72) |  | 1.42 | (-0.21, 3.04) |
|  | GSS 2010 |  | 2.18 | (1.04, 4.57) |  | 2.91 | (1.10, 7.70) |  | -2.00 | (-5.79, 1.80) |  | 0.36 | (-1.79, 2.51) |
|  | GSS 2012 |  | 1.38 | (0.63, 3.02) |  | 2.41 | (0.49, 11.85) |  | -4.67 | (-7.10, -2.25) |  | 1.54 | (-0.27, 3.35) |
|  | **Pooled estimate** |  | **1.25** | **(0.91, 1.70)** |  | **2.11** | **(1.17, 3.80)** |  | **-2.55** | **(-3.51, -1.59)** |  | **0.81** | **(0.09, 1.53)** |
|  |  |  |  |  |  |  |  |  |  |  |  |  |  |
| **European/Other** | NZHS 2002/03 |  | 1.43 | (0.53, 3.88) |  |  |  |  |  |  |  |  |  |
|  | NZHS 2006/07 |  | 1.27 | (0.83, 1.93) |  |  |  |  | -1.57 | (-2.48, -0.66) |  | -0.82 | (-1.98, 0.35) |
|  | NZHS 2011/12 |  | 0.64 | (0.31, 1.34) |  |  |  |  | -2.72 | (-4.10, -1.33) |  | -0.97 | (-2.72, 0.78) |
|  | GSS 2008 |  | 0.93 | (0.27, 3.22) |  | 2.49 | (1.60, 3.87) |  | -4.26 | (-5.99, -2.53) |  | -1.20 | (-2.81, 0.41) |
|  | GSS 2010 |  | 2.18 | (1.04, 4.57) |  | 1.94 | (1.09, 3.47) |  | -3.93 | (-6.48, -1.39) |  | -1.91 | (-3.77, -0.05) |
|  | GSS 2012 |  | 1.38 | (0.63, 3.02) |  | 2.83 | (1.71, 4.69) |  | -4.71 | (-6.87, -2.56) |  | -1.06 | (-2.96, 0.84) |
|  | **Pooled estimate** |  | **1.25** | **(0.91, 1.70)** |  | **2.44** | **(1.83, 3.26)** |  | **-3.23** | **(-4.53, -1.92)** |  | **-1.11** | **(-1.81, -0.40)** |
